# Maternal pre-pregnancy BMI, offspring epigenome-wide DNA methylation, and childhood obesity: findings from the Boston Birth Cohort

**DOI:** 10.1186/s12916-023-03003-5

**Published:** 2023-08-23

**Authors:** Jiahui Si, Anat Yaskolka Meir, Xiumei Hong, Guoying Wang, Wanyu Huang, Colleen Pearson, William G. Adams, Xiaobin Wang, Liming Liang

**Affiliations:** 1 Departments of Epidemiology and Biostatistics, Harvard T.H. Chan School of Public Health, Boston, MA USA; 2 Center On the Early Life Origins of Disease, Department of Population, Family and Reproductive Health, Johns Hopkins Bloomberg School of Public Health, Baltimore, MD USA; 3 Department of Civil and Systems Engineering, Johns Hopkins University Whiting School of Engineering, Baltimore, MD USA; 4 Department of Pediatrics, Boston University Chobanian & Avedisian School of Medicine and Boston Medical Center, Boston, MA USA; 5 Department of Pediatrics, Johns Hopkins School of Medicine, Baltimore, MD USA

**Keywords:** DNA methylation, Childhood Obesity, Birthweight, Maternal pre-pregnancy obesity, Early life origins of chronic diseases, Boston Birth Cohort

## Abstract

**Background:**

Maternal pre-pregnancy obesity is an established risk factor for childhood obesity. Investigating epigenetic alterations induced by maternal obesity during fetal development could gain mechanistic insight into the developmental origins of childhood obesity. While obesity disproportionately affects underrepresented racial and ethnic mothers and children in the USA, few studies investigated the role of prenatal epigenetic programming in intergenerational obesity of these high-risk populations.

**Methods:**

This study included 903 mother–child pairs from the Boston Birth Cohort, a predominantly urban, low-income minority birth cohort. Mother-infant dyads were enrolled at birth and the children were followed prospectively to age 18 years. Infinium Methylation EPIC BeadChip was used to measure epigenome-wide methylation level of cord blood. We performed an epigenome-wide association study of maternal pre-pregnancy body mass index (BMI) and cord blood DNA methylation (DNAm). To quantify the degree to which cord blood DNAm mediates the maternal BMI-childhood obesity, we further investigated whether maternal BMI-associated DNAm sites impact birthweight or childhood overweight or obesity (OWO) from age 1 to age 18 and performed corresponding mediation analyses.

**Results:**

The study sample contained 52.8% maternal pre-pregnancy OWO and 63.2% offspring OWO at age 1–18 years. Maternal BMI was associated with cord blood DNAm at 8 CpG sites (genome-wide false discovery rate [FDR] < 0.05). After accounting for the possible interplay of maternal BMI and smoking, 481 CpG sites were discovered for association with maternal BMI. Among them 123 CpGs were associated with childhood OWO, ranging from 42% decrease to 87% increase in OWO risk for each SD increase in DNAm. A total of 14 identified CpG sites showed a significant mediation effect on the maternal BMI-child OWO association (FDR < 0.05), with mediating proportion ranging from 3.99% to 25.21%. Several of these 14 CpGs were mapped to genes in association with energy balance and metabolism (*AKAP7*) and adulthood metabolic syndrome (*CAMK2B*).

**Conclusions:**

This prospective birth cohort study in a high-risk yet understudied US population found that maternal pre-pregnancy OWO significantly altered DNAm in newborn cord blood and provided suggestive evidence of epigenetic involvement in the intergenerational risk of obesity.

**Supplementary Information:**

The online version contains supplementary material available at 10.1186/s12916-023-03003-5.

## Background

The prevalence of childhood obesity was 19.7% and affected about 14.7 million children and adolescents in the USA in 2017–2020 [1], increasing from 18.5% in 2015–2016 [2]. Childhood obesity is more common in racial minority children and has increased dramatically [3]. Obesity remains a serious public health threat that disproportionately affects racial minority children [4].

Maternal pre-pregnancy obesity is an established risk factor for offspring cardiometabolic dysfunction, including obesity [5]. Given that 25% of conceptions occur among obese women in the USA [6], maternal obesity before pregnancy is one of the most common intrauterine risk factors for childhood obesity. However, there is still limited mechanistic insight, especially in racial minority children. One plausible hypothesis points to epigenetic modification, as mouse models demonstrated the induction of epigenetic alterations by maternal obesity can affect the later risk of obesity in offspring [7–9].

However, evidence from prospective birth cohort studies connecting maternal BMI, fetal DNAm, and childhood BMI remains limited [10]. Only five studies explored the association of maternal BMI with genome-wide DNAm in newborn cord blood [11–15]. The majority of previous studies were based on Illumina HumanMethylation27 BeadChip [11] or 450 BeadChip [12–14]. Notably, none of the previous findings overlapped with one another in terms of gene level. Few studies conducted on African Americans were limited by a small sample size [11, 14]. In addition, previous studies did not consider other maternal lifestyle factors like smoking, which may exacerbate the adverse DNAm alterations induced by maternal BMI, and such evidence is lacking. Most notably, there is a lack of longitudinal follow-up in children from birth to adolescence to estimate the impact of maternal pre-pregnancy BMI-related CpG methylation on offspring obesity.

To fill this gap, we examined the association between maternal pre-pregnancy BMI and epigenome-wide methylation of cord blood-derived DNA (using the latest Illumina Infinium MethylationEPIC BeadChip with more than 850,000 methylation sites) among mother–child pairs enrolled in the Boston Birth Cohort (BBC), a predominantly urban, low-income ethnic minority (Black and Hispanic) sample in Boston, MA. We then investigated the relationships between the identified maternal BMI-associated methylation sites and birthweight/child overweight or obesity (OWO) from 1 to 18 years old and further identified potential pathways by causal mediation analysis.

## Methods

### Study population

The present study included 903 mother–child pairs from the BBC. The BBC, a predominantly urban, low-income minority birth cohort, was initiated in 1998 with a rolling enrolment in Boston, MA. Briefly, mothers who delivered singleton live births at the Boston Medical Center (BMC) were invited to participate shortly after giving birth. Twins or triplets and newborns with major birth defects were excluded. The BBC study was designed to over-sample low birthweight (< 2.5 kg) and preterm (< 37 weeks of gestation) births at enrollment. All enrolled mothers completed a standard questionnaire interview to assess maternal socio-demographic characteristics, lifestyle (including smoking, alcohol consumption, and dietary habits), and reproductive and medical history.

Cord blood samples were obtained at delivery by trained nursing staff of the labor and delivery (L&D) service. Blood samples were then immediately stored in a designated − 20° freezers in L&D for study collection. Cord blood was centrifuged (0° Celsius for 13 min) and fractionated into plasma, white blood cells, and red blood cells. All biospecimens are stored in − 80° freezers pending analysis.

Clinical information on mothers and newborns, including birth outcomes, was obtained from their medical records. Beginning in 2004 under a separate but linked IRB protocol, maternal-child pairs were invited to continue their participation in a postnatal follow-up study if the child continued primary or specialty care at BMC. A standardized questionnaire was used to assess postnatal demographic, infant feeding, and environmental information. The study design, data collection methods, and long-term follow-up have been described and published previously [16].

The study protocol has received initial and annual continuation approval by the Institute Review Boards of Boston Medical Center and the Johns Hopkins Bloomberg School of Public Health. All study mothers provided written informed consent. Study children are not able to give true informed consent legally until they turn 18. Thus, we obtained assent from study children at BMC IRB determined ages. Written informed consent was obtained when the child reached age 18.

### DNAm profiling in cord blood

DNA samples were isolated from EDTA-treated peripheral white blood cells and shipped to the University of Minnesota Genomics Center for methylation profiling. The quality of the DNA samples has been demonstrated in our previous study using the Illumina BeadChip [17]. Detailed quality control process of the DNA methylation data has been previously published [18]. Briefly, DNA methylation was measured for 963 cord blood samples (plus 21 replicates) using the Illumina Infinium MethylationEPIC BeadChip (850K). This platform interrogates a total of 865,859 CpG sites. Both cord blood samples and maternal samples (*n* ~ 420 samples) were randomly placed in each 96-well DNA plate during the DNA methylation profiling. The laboratory staff were blinded to the sample placement. Only methylation data measured in cord blood was used in the present study.

*β*-value for each CpG site was reported, ranging from 0 to 1, to signify the percentage of DNAm at each CpG site. We used minfi package [19] to perform existing analytic pipelines. Probes were excluded if they had bead count < 3 in 5% of samples or had > 5% of samples with a detection *P* > 0.01 (*n* = 4193) and had an annotated SNP at the measured or extension site or that overlapped with the probe or that potentially cross-hybridized to other genomic locations [20] (*n* = 140,271). Samples were excluded due to the following reasons: if they (1) were outliers detected by multidimensional scaling (MDS) analysis or a median log_2_ intensity value < 10 (*n* = 13); (2) were sex mixed-up samples (*n* = 7); (3) had missing rate > 0.02 across probes (*n* = 1).

Furthermore, MDS plots confirmed male cord blood samples, female cord blood samples, and maternal blood samples, which clustered separately, as expected. Correlations for the duplicates were computed among the 21 pairs of duplicates. The correlation for duplicate measurements on the same sample ranged from 0.990 to 0.996. We then performed the single-sample Noob (ssNoob) methods for background and dye bias correction [21] and performed quantile normalization to normalize type 1 and type II probes. We further excluded 39 mother–child pairs with missing maternal BMI data. These filtering process resulted in 903 samples with 721,395 CpG sites for the downstream analyses. Additional file 1: Table S1 presents the characteristics of the 903 pairs enrolled in this study compared to the remaining recruited mother–child pairs (*n* = 7720) in the BBC. The key variables were comparable between the 903 pairs and the remaining ones.

### Assessment of perinatal and postnatal variables

Maternal pre-pregnancy weight and height assessments were based on maternal questionnaire interviews at enrollment. Maternal BMI was calculated as weight in kilograms divided by the square of height in meters and then categorized into two groups: non-OWO (< 25 kg/m^2^) and OWO (≥ 25 kg/m^2^). Maternal smoking during pregnancy was defined according to the following questions:(1) in the 6 months before you found out you were pregnant, did you smoke/use tobacco? (2) did you smoke/use tobacco in the first three months of pregnancy? (3) did you smoke/use tobacco in the middle 3 months of pregnancy? and (4) did you smoke/use tobacco in the last 3 months of pregnancy. We defined ever-smoking during pregnancy mothers as those who answered “yes” to any of the above questions and defined others as never-smoking during pregnancy mothers.

Birthweight was abstracted from the electronic medical record (EMR). Other important covariates included: child’s sex, maternal age at delivery, maternal race (Black, White, or Hispanic), maternal education level (high school or less, versus others), maternal alcohol consumption (ever drinking alcohol during pregnancy versus others), and parity (not including the index pregnancy, zero versus one or more). Gestational age was determined using an established algorithm based on both the last menstrual period and the result of early ultrasound (< 20 weeks of gestation) to maximize accuracy and minimize missing data. Details of the covariates can be found elsewhere [17].

### Assessment of overweight or obesity (OWO) in childhood

Child weight and height were measured by medical staff during pediatric well-child visits and retrieved from the EMR. Children’s repeated measurements of BMI from birth to age 18 were collected. The first measure was used if repeated measures were taken within one year of age. BMI *z*-scores and percentiles for each age-window (1 year of age) were calculated based on US national reference data by age and gender [22]. Missing percentiles were imputed using the average of the last and following observed values. OWO was defined as BMI ≥ 85th percentile of age and gender.

### Statistical analysis

**Fig. 1:**
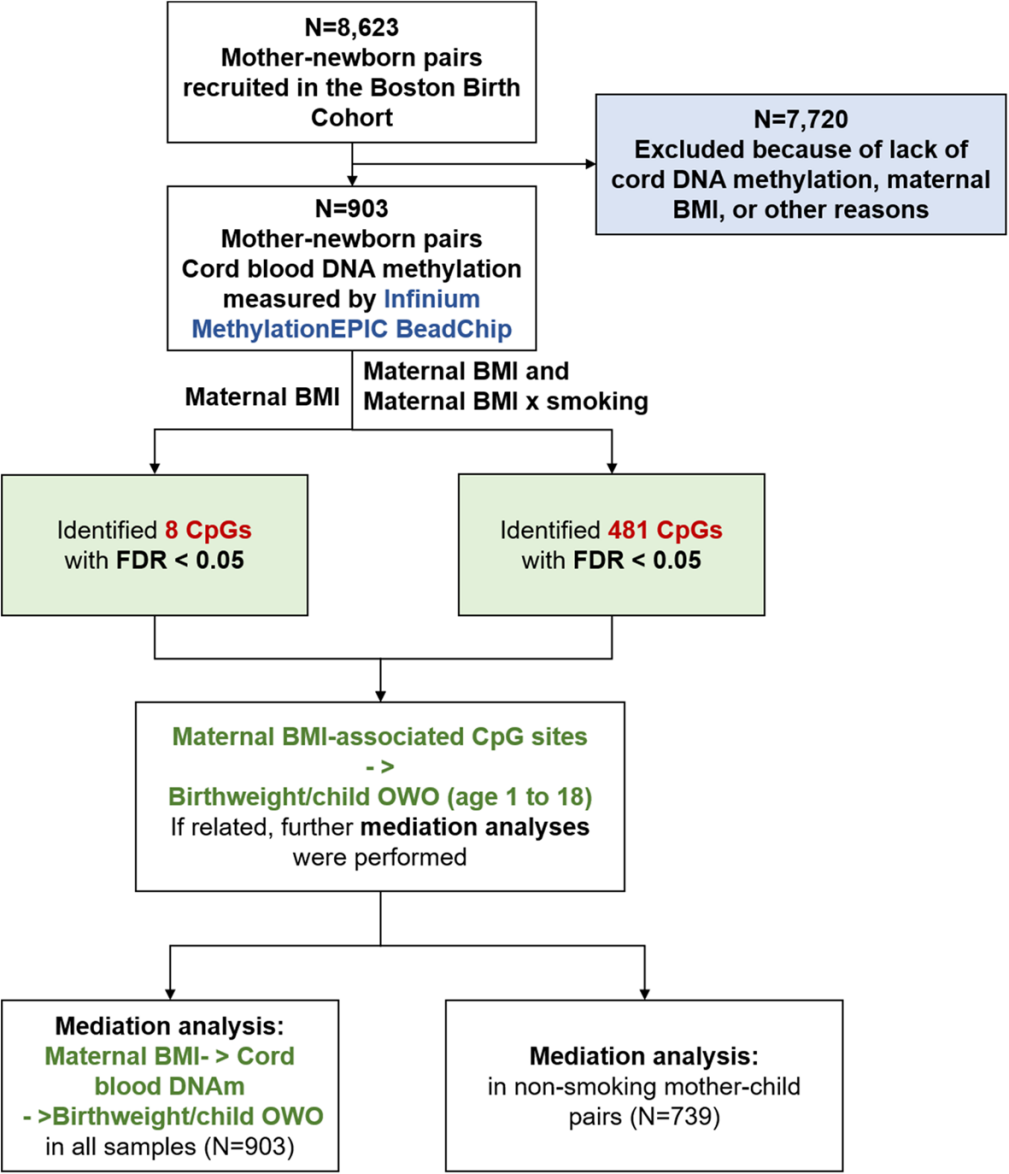
Analyses flowchart of the present study

Figure 1 presented the overall analysis flowchart. We compared the demographic and clinical characteristics of newborns exposed to maternal pre-pregnancy OWO versus those who were not, with chi-square test for categorical variables and ANOVA for continuous variables. We used false discovery rate (FDR) [23] < 0.05 to determine epigenome-wide significant CpGs in relation to maternal BMI.

### Differentially methylated sites associated with maternal BMI

Linear regression was applied to investigate differentially methylated sites in cord blood that were associated with maternal BMI, with methylation values as dependent variables, maternal BMI as the independent variable, and child’s sex, maternal age at delivery, maternal race, maternal education level, maternal alcohol consumption, parity, and gestational age as covariates. We also adjusted for estimated cord blood cell composition (CD4 + , CD8 + T cells, B cells, monocytes, granulocytes, natural killer cells, and nucleated red blood cells). The cell type proportion was inferred for each cord blood sample, based on external cord blood reference DNA methylation signatures of the constituent cell type from the Illumina Infinium Methylation450 BeadChip by the minfi package [24]. To quantify latent factors, including the effects of unobserved batch effects and other unmeasured confounding factors, we used smart surrogate variable (SV) analysis by the smartSVA package [25]. A total of 54 SVs were generated and included as covariates in the above model.

To account for the possible interplay of maternal BMI and smoking in impacting the methylation level, we performed likelihood ratio test comparing model 1 (with maternal BMI and the cross-product term of maternal BMI x smoking) to model 2 (without these two terms).

The CpG sites associated with pre-pregnancy maternal BMI were annotated to the corresponding genes based on an official EPIC array annotation file from Illumina [26]. Further enrichment analyses were conducted using the Genetic Association Database (GAD) enclosed within the Database for Annotation, Visualization and Integrated Discovery (DAVID) (https://david.ncifcrf.gov/).

### Mediation analyses

As a first step, we performed multiple linear regression (for birthweight) or logistic regression (for child OWO) analyses and identified whether the maternal BMI-associated CpG sites (identified in EWAS) were associated with newborn birthweight and child OWO. Covariates adjusted included maternal BMI, smoking, maternal age at delivery, race, education level, alcohol consumption, child’s sex, parity, gestational age, cord blood cell compositions, and all SVs. In the analyses of child OWO, we additionally adjusted for newborn birthweight.

The subsequent analyses focused on CpG sites associated with both maternal BMI and newborn birthweight or child OWO (*P* < 0.05). Using parametric regression models, we performed causal mediation analysis, achieved by the R “mediation” package [27]. Two models were estimated for each CpG: (1) a model for the mediator (*β*-value for each CpG site as a continuous variable) conditional on exposure (maternal BMI) and covariates (maternal smoking, age at delivery, race, education level, and alcohol consumption, child’s sex, parity, gestational age, cord blood cell compositions and all SVs); (2) a model for the outcome (newborn birthweight or child OWO) conditional on exposure, the mediator, and covariates. We allowed for the presence of exposure-mediator interactions in the outcome regression model. In our non-smoking restricted analysis, we removed the smoking covariate from the model.

We aimed to calculate how much maternal BMI association with child OWO risk (average total effect) was attributable to the mediating effect of methylation level at a specific locus (average causal mediation effect, ACME). The proportion attributable to the ACME was calculated as ACME divided by the total effect on the log odds scale, with 0 indicating no mediation effect. Stratified mediation analyses by maternal smoking status during pregnancy were also performed. We adjusted for multiple testing by the FDR method. All analyses were conducted using R software, version 3.4.1 (R Project for Statistical Computing).

## Results

A total of 903 mother–child pairs were included after data quality control steps; 641 mothers (71.0%) were Black; 478 (52.9%) children were boys. The median value of pre-pregnancy BMI was 26.62 kg/m^2^, with the interquartile range being 7.86 kg/m^2^. The study sample contained 52.8% maternal pre-pregnancy OWO and 63.2% offspring OWO at age 1–18 years, respectively. Compared to non-OWO mothers, OWO mothers were more likely to be older, Black, and had a higher proportion of smokers. The children born to OWO mothers had a higher prevalence of OWO from ages 1 to 18 years (Table 1).

**Table 1:** Characteristics of mothers-child pairs in total sample and stratified by maternal pre-pregnancy overweight or obesity (OWO)

	Total sample	Maternal OWO status		*P* value
		Non-OWO	OWO	
**Maternal characteristics**				
*N*	903	426	477	-
Maternal age (years)	28.28 (6.59)	27.08 (6.54)	29.35 (6.45)	< 0.001
Maternal race				< 0.001
Black	641 (71.0)	280 (65.7)	361 (75.7)	
White	51 (5.7)	24 (5.6)	27 (5.7)	
Hispanic	211 (23.4)	122 (28.6)	89 (18.7)	
Maternal body mass index (kg/m^2^)	26.95 (6.46)	21.98 (2.01)	31.40 (5.79)	-
Alcohol drinking during pregnancy	75 (8.3)	29 (7.0)	46 (10.0)	0.124
Ever smoking during pregnancy	164 (18.2)	65 (15.3)	99 (20.8)	0.033
Maternal education level (> high school)	304 (33.7)	150 (35.2)	154 (32.3)	0.346
**Child’s characteristics**				
Gender (male)	478 (52.9)	231 (54.2)	247 (51.8)	0.463
Gestational age (weeks)	38.62 (2.47)	38.75 (2.13)	38.47 (2.77)	0.100
Parity (> = 1 live birth)	500 (55.4)	203 (47.7)	297 (62.3)	< 0.001
Birthweight (g)	3128 (666)	3115 (587)	3140 (729)	0.572
OWO at age 1–18 years^a^	571 (63.2)	233 (54.7)	338 (70.8)	< 0.001
Obesity at age 1–18 years^b^	390 (43.2)	155 (36.4)	235 (49.3)	< 0.001
Obesity at age 2–5 years	282 (31.2)	108 (25.4)	174 (36.5)	< 0.001
Obesity at age 6–11 years	311 (34.4)	111 (26.1)	200 (41.9)	< 0.001
Obesity at age 12–18 years	287 (31.8)	101 (23.7)	186 (39.0)	< 0.001

Data are presented as mean ± SD, or number (%)

^a^Children who were defined as OWO (body mass index ≥ the 85th percentile of age and gender) at any of the ages from 1 to 18 years

^b^Children who were defined as obesity (body mass index ≥ the 95th percentile of age and gender) at any of the ages from 1 to 18 years

### Epigenome-wide association analysis of maternal BMI in cord blood

**Fig. 2:**
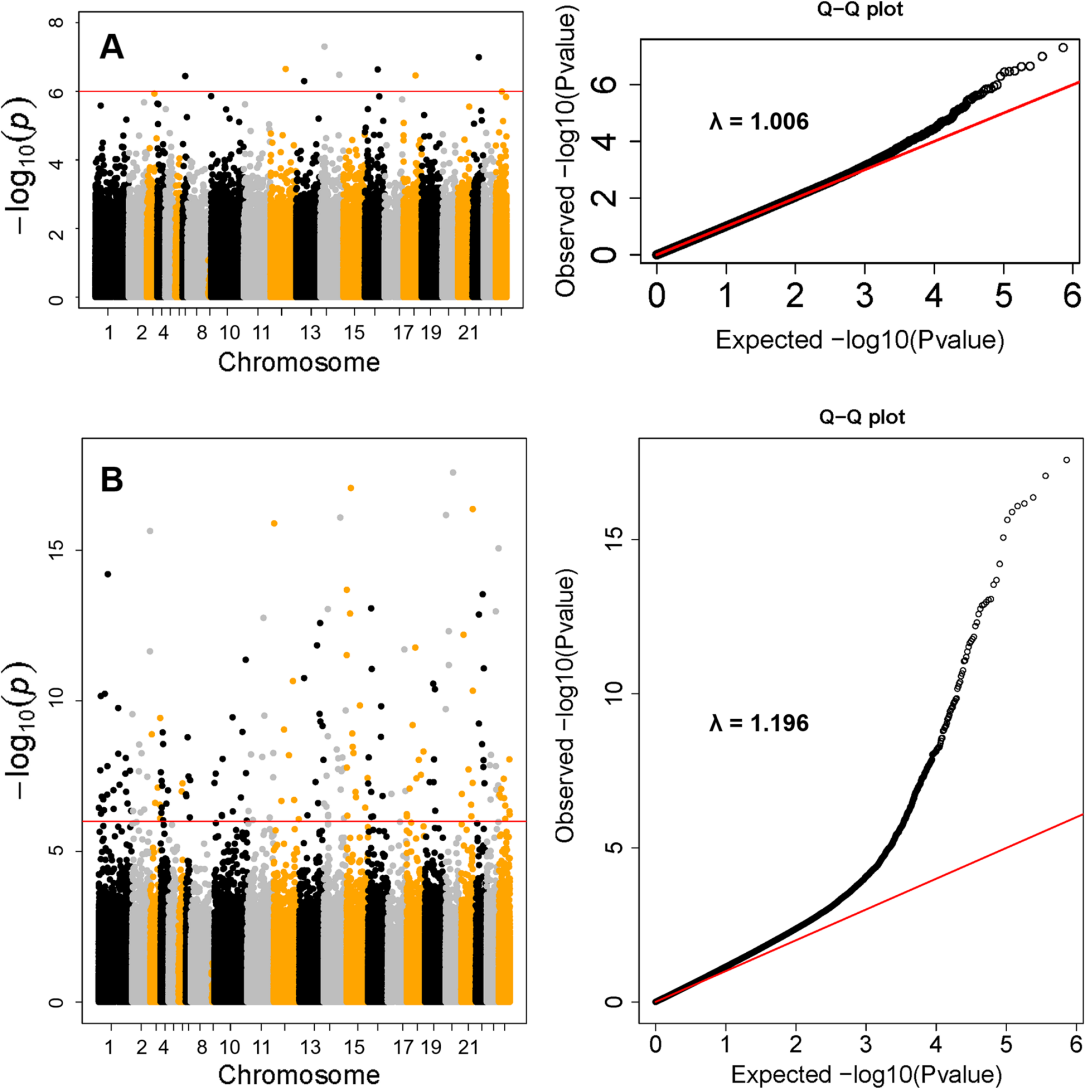
Manhattan plot and Q.Q. plot of the *P* values of the associations between maternal body mass index (BMI) (**A**), maternal BMI and maternal BMI x maternal smoking (**B**) and each cytosine-phosphoguanine (CpG) site. **A** The associations without accounting for maternal BMI x maternal smoking interaction on DNAm. **B** The associations accounting for maternal BMI x maternal smoking interaction on DNAm. We performed likelihood ratio test (2-df LRT) comparing model 1 (with maternal BMI and the cross-product term of maternal BMI x smoking) to model 2 (without these two terms). In the Manhattan plot, the red line represents − log10 *P* at false discovery rate (FDR) = 0.05

We identified 8 CpG sites, corresponding to 6 genes, associated with maternal BMI at genome-wide significance (FDR < 0.05) (Table 2 and Fig. 2). Figure 2a shows the Manhattan plot and the Quantile–Quantile plot of the association between maternal BMI and cord blood DNAm. The genomic inflation factor was 1.01 indicating little residual confounding. The maximum adjusted hypermethylated difference (standard error, SE) per one unit increase in maternal pre-pregnancy BMI was 0.00026 (4.89E − 05) for cg21701395, which was annotated to *TP53INP1* (*P* = 2.31E − 07). The corresponding maximum hypomethylated adjusted difference (SE) was − 0.00067 (1.30E − 04) for cg10272744, which was annotated to *LL22NC01-81G9.3* (*P* = 3.58E − 07). Other significant hypermethylated CpG sites were identified in the *PTEN* and *KILLIN* genes as indicated by the positive coefficients in relation to maternal BMI. The CpG sites in the *AAGAB*, *ALPK1*, and *ERCC8* genes were significantly hypomethylated in newborns born to mothers with higher BMI as indicated by the negative coefficients. When further dividing mothers into three groups (under/normal weight, overweight, and obesity), we observed obesity mothers having the highest DNA methylation level at cg13694461 and cg06466203, lowest at cg02266725, cg10272744, and cg23151800 (Additional file 2: Fig. S1). Accounting for the possible interplay of maternal BMI and smoking in impacting the methylation level, we performed a likelihood ratio test and identified 481 CpG sites with altered DNAm in newborns that were associated with maternal BMI (FDR < 0.05). Additional genomic information on these CpG sites was provided in Additional file 3: Table S2. Manhattan plot indicated that significant associations were distributed across the genome (Fig. 2b). The elevated tail of the QQ-plot indicated enrichment of robust association and the genomic inflation factor 1.19 indicated small residual confounding or polygenic effect. Enrichment analysis of the 481 CpG sites revealed that their annotated genes were enriched in genes associated with triglycerides, hematocrit, and tobacco use disorder (FDR < 0.05; Additional file 1: Table S3). We identified ten genes with at least two significantly altered DNAm sites: *DIP2C*, *ADARB2*, *LAMA3*, *MAP4K4*, *MCEE*, *PLEKHG1*, *PTPRN2*, *RIN3*, *SMTNL2*, and *THSD4*.

**Table 2:** Epigenome-wide DNA methylation association study identified 8 CpG sites significantly associated with maternal pre-pregnancy body mass index in 903 mother–child pairs from the Boston Birth Cohort

Chr	CpG	Gene	Relation to Gene	EWAS		
				*β* (SE)^a^	*P*	FDR
6	cg13694461	*(HLA-E*^*b*^*)*	/	0.00012 (2.09E − 05)	4.94E − 08	0.036
15	cg22940988	*AAGAB*	Body	− 0.00032(6.04E − 05)	1.01E − 07	0.037
4	cg02266725	*ALPK1*	Body	− 0.00036 (6.80E − 05)	2.23E − 07	0.037
10	cg06466203	*PTEN* *KILLIN;*	5'UTR TSS200	0.00018 (3.50E − 05)	3.44E − 07	0.037
22	cg10272744	*LL22NC01-81G9.3*	TSS1500	− 0.00067 (1.30E − 04)	3.58E − 07	0.037
8	cg21701395	*TP53INP1*	5'UTR	0.00026 (4.89E − 05)	2.31E − 07	0.037
6	cg22307152	*(LINC01625*^*b*^*)*	/	− 0.00032 (6.12E − 05)	3.27E − 07	0.037
5	cg23151800	*ERCC8*	Body	− 0.00050 (9.78E − 05)	5.03E − 07	0.046

Linear regression was fitted with adjustment for child’s sex, maternal age at delivery, maternal race, maternal education level, maternal alcohol consumption, parity, gestational age, estimated cord blood cell composition (CD4 + , CD8 + T cells, B cells, monocytes, granulocytes, natural killer cells, and nucleated red blood cells), and all surrogate variables. CpG = cytosine-phosphoguanine site; Chr = chromosome; EWAS = epigenome-wide association; SE = standard error; FDR = false discovery rate; TSS200 = within 200 bp from transcription start site; TSS1500 = within 1500 bp from transcription start site; Body = the CpG is in gene body; and UTR = untranslated region

^a^Effect sizes were calculated based on normalized methylation values, denoting the methylation difference per unit increase of maternal BMI

^b^For inter-genic CpG sites, we used the UCSC Genome Browser on Human (GRCh37/hg19) to locate the nearest annotated gene

### Maternal BMI-associated CpGs and birthweight

Of the total 481 maternal BMI-associated CpG sites, 26 were associated with newborn birthweight in this study (*P* value < 0.05). The adjusted differences in newborn birthweight per 1 SD increment of cord blood DNAm ranged from -92.5 g for cg04107082 (*P* = 0.031) to 64.9 g for cg26779585 (*P* = 0.003). Then, out of the total 26 CpGs, we estimated the mediation effect of each single CpG on the maternal BMI-birthweight association. After correction for multiple testing, we did not detect any significant mediation effect (Additional file 1: Table S4).

### Maternal BMI-associated CpGs and child OWO

Additional file 1: Table S5 presents the associations between the identified 481 maternal BMI-associated CpG sites and child OWO, with adjustment for maternal BMI, birthweight, and other covariates. A total of 123 CpGs showed a significant association with child OWO in at least one age-window (*P* value < 0.05). The adjusted odds ratio (ORs) per 1 SD increment of cord blood DNAm ranged from 0.58 for cg07686439 (*P* = 0.005, OWO at 3 years old) to 1.87 for cg08469255 (*P* = 0.015, OWO at 4 years old).

In the following mediation analysis, we focused on the CpGs in association with child OWO, and found a total of 14 CpG sites showed a significant mediation effect on the maternal BMI-child OWO association in at least one age group (FDR < 0.05) (Additional file 2: Fig. S2). As presented in Table 3, 21.69%, 17.41%, and 14.88% of the maternal BMI-associated child OWO risk at age 3 years were mediated through methylation levels at cg02059896 (*P* < 0.001), cg14257335 (*P* = 0.004), and cg15059222 (*P* = 0.003), respectively. A total of 8 CpG sites mediated the association of maternal BMI with child OWO at age 7–9 years, with the mediating proportions ranging from 4.38% (cg06292624, *P* = 0.007) to 25.21% (cg14015044, *P* = 0.009). At the time window of age 13 years, we also observed 5 CpG sites mediating the association of maternal BMI with child OWO. The mediating proportions ranged from 3.99% at cg06292624 (*P* = 0.012) to 10.57% at cg08391482 (*P* = 0.011).

**Table 3:** Mediation effect of CpG sites on the maternal BMI-child overweight or obesity (OWO) association in 903 mother–child pairs and 739 non-smoking during pregnancy mother–child pairs from the Boston Birth Cohort

CpG	Gene	Outcome	Whole sample (*N* = 903)		Subset without maternal smoking (*N* = 739)	
			% Mediated	*P*	% Mediated	*P*
cg06292624	*AKAP7*	OWO at 13 years	3.99	0.011	3.18	0.048
		OWO at 9 years	4.38	0.007	3.52	0.047
		OWO at 8 years	5.00	0.004	3.91	0.044
		OWO at 7 years	5.04	0.007	4.40	0.038
cg17886022	*LINC00174*	OWO at 13 years	8.78	0.005	9.95	0.033
		OWO at 9 years	9.36	0.002	10.71	0.019
		OWO at 8 years	9.17	0.005	11.29	0.015
		OWO at 7 years	8.98	0.010	11.53	0.022
**cg04347874**	***NKX2-1***	**OWO at 9 years**	**8.58**	**0.005**	**0.43**	**0.610**
		**OWO at 8 years**	**8.17**	**0.011**	**0.35**	**0.630**
		**OWO at 7 years**	**8.94**	**0.005**	**0.60**	**0.600**
cg04390217	*PECR*	OWO at 13 years	5.95	0.006	2.60	0.080
		OWO at 9 years	5.14	0.011	2.10	0.100
		OWO at 8 years	5.30	0.013	2.01	0.120
**cg09545197**	***KCNA3***	**OWO at 8 years**	**13.35**	**0.016**	**3.13**	**0.180**
		**OWO at 7 years**	**15.83**	**0.007**	**3.89**	**0.170**
cg13331383	*TMEM203*	OWO at 7 years	5.66	0.016	8.68	0.036
	*NDOR1*	OWO at 5 years	8.22	< 0.001	11.53	0.001
cg04383707	*NCCRP1*	OWO at 13 years	4.67	0.006	2.14	0.110
**cg08391482**	***SERBP1***	**OWO at 13 years**	**10.57**	**0.010**	**1.25**	**0.250**
cg01886035	*CGREF1*	OWO at 9 years	4.32	0.009	1.07	0.320
cg14015044	*TNFRSF10C*	OWO at 8 years	25.21	0.010	30.75	0.010
cg23732384	*MAP2K7*	OWO at 7 years	10.19	0.016	15.80	0.003
**cg02059896**	**(*PIKFYVE***^a^**)**	**OWO at 3 years**	**21.69**	** < 0.001**	**1.64**	**0.490**
cg14257335	*KLHL22*	OWO at 3 years	17.41	0.004	19.79	0.002
**cg15059222**	***CAMK2B***	**OWO at 3 years**	**14.88**	**0.003**	**2.80**	**0.150**

All models adjusted for maternal age, education level, race, parity, smoking (only in analysis among whole mother–child pairs), alcohol consumption, gestational age, child sex, birthweight, cord blood cell compositions (CD8, CD4, N.K., B cell, monocytes, granulocytes, nucleated red blood cells), and all surrogate variables

^a^For inter-genic CpG sites, we used the UCSC Genome Browser on Human (GRCh37/hg19) to locate the nearest annotated gene

To test whether the mediation effect differs among smoking and non-smoking participants, we restricted the mediation analyses to never-smoking during pregnancy mother–child pairs (*n* = 739). Of note, we did not observe a significant mediation effect for cg04347874, cg09545197, cg08391482, cg02059896, and cg15059222 in the non-smoking group. The main mediation results for the overall sample appeared to be driven by smoking in these mother–child pairs for these 5 CpG sites (Table 3). The rest of CpG sites tended to have comparable mediating effects on the maternal BMI-child OWO association in both groups.

## Discussion

In a population of predominantly Black mother–newborn pairs who are low-income and living in disinvested communities in Boston, Massachusetts, we identified 481 differentially methylated CpG sites in the newborn cord blood associated with maternal pre-pregnancy BMI. This study extends previous work based on the Illumina Infinium Methylation27 or 450 BeadChip, which were biased toward promoter regions and missed more dynamic methylation sites, including the enhancers. To the best of our knowledge, this is the first EWAS using the latest Illumina Infinium MethylationEPIC BeadChip and the largerst EWAS in a racial minority population and the first prospective study to link maternal BMI-associated cord blood DNA methylation with children’s BMI from birth to adolescence. Further mediation analyses revealed multiple CpG sites mediated the impact of maternal BMI on offspring obesity, making contributions to understanding intergenerational transmission of overweight and obesity.

Childhood obesity prevalence in the US was 12.7, 20.7, and 22.2% among 2–5, 6–11, and 12–19 years old in 2017–2020, respectively [1]. The percentage of obese offspring in the present study was 31.4, 34.7, and 32.0% among the age group of 2–5, 6–11, and 12–18 years old. The BBC cohort mainly recruited low-income Hispanic and non-Hispanic Black mother–child pairs, which have the highest rates of obesity in the US. Compared to the cross-sectional study design, we can capture more obese children through repeated measurements across the pediatric age range.

In our EWAS of maternal pre-pregnancy BMI and cord blood DNA methylation, we replicated several associations at the gene level reported in previous studies: *PRR16* [11], *PTPRN2*, *SEPT9*, *XXYLT1* [13], and *GFI1* [14]. Some of these findings were supported by existing literature and biologically plausible. For example, *PTPRN2* encodes a protein involved in regulating insulin secretion [28]. The specific substrates and binding partners suggest its function for metabolism, including obesity and type 2 diabetes [29, 30]. We identified two CpGs of *PTPRN2* gene with significant association with maternal BMI. A previous case–control study (252 obese children and 386 controls) showed an epigenetic association of *PTPRN2* gene with childhood obesity under certain genetic backgrounds [31]. *PTPRN2* has been reported in relation to BMI in GWAS [32–34]. Together with our result, these literatures suggest that the methylation level of *PTPRN2* might be on the pathway from maternal BMI to offspring obesity.

**Fig. 3:**
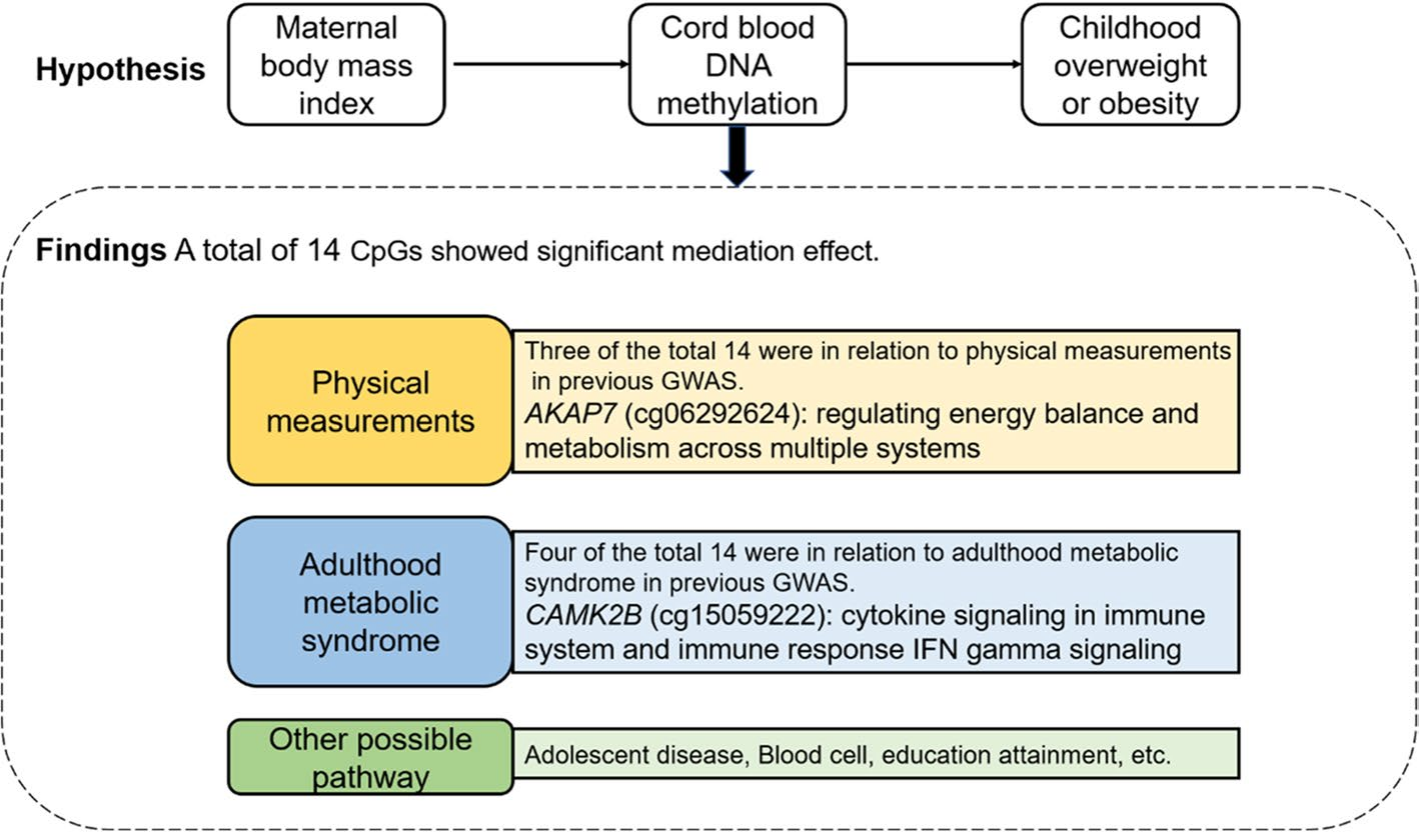
The diagram of possible pathway from maternal body mass index to childhood overweight or obesity through DNA methylation identified in the present study

For the first time, we quantitatively estimated how much of the effects of maternal BMI on offspring long-term obesity risk are mediated through DNAm alterations in newborn cord blood. The mediated proportions were up to > 25% (cg14015044, *TNFRSF10C*). We summarized the annotated or nearest annotated gene of the identified CpGs with significant mediation effects in our study and the previous GWAS findings (Fig. 3 and Additional file 1: Table S6) [33–58]. Three identified CpG sites were linked to physical measurements in previous GWAS studies. CpG cg06292624 was located at the gene *AKAP7*, a member of scaffolding proteins that bind to protein kinase A (PKA), which plays a vital role in intracellular PKA-dependent signaling pathways [59]. Both mouse and human models demonstrated a central role for the PKA signaling pathway in regulating energy balance and metabolism across multiple systems [60]. Previous GWAS identified that the *AKAP7* SNPs were significantly associated with body weight [34], height [33], and hip circumference adjusted for BMI [61]. The methylation level at CpG cg06292624 was found to be associated with the risk of child OWO in our study. Further mediation analysis showed that ~ 5% of the offspring obesity risk related to maternal BMI was mediated through methylation at cg06292624, suggesting that such epigenetic regulation related to maternal BMI might influence the subsequent risk of obesity in offspring.

Four of the total 14 identified CpGs mediating the association between maternal BMI and child’s OWO were mapped to genes that have been reported in association with adulthood metabolic syndrome in previous GWAS studies. CpG cg15059222 maps to the *CAMK2B* (Calcium/Calmodulin-Dependent Protein Kinase Type II Beta Chain) gene. Among its related pathways are cytokine signaling in the immune system and the immune response IFN gamma signaling pathway [62]. The perturbation of this process may lead to impaired homeostatic responses and possibly disease states, including obesity and diabetes [63, 64]. The methylation level at CpG cg15059222 was found to be associated with the risk of obesity at 3 years old in our study. Further mediation analysis noted that more than 14% of the increased risk of early-onset (at age 3 years) OWO related to maternal pre-pregnancy BMI was mediated through methylation level at cg15059222. SNPs in *CAMK2B* have been consistently linked to birthweight and risk of type 2 diabetes in different populations [34, 50–57]. Taken together, maternal BMI-induced epigenetic modification of DNA may play an important part in the underlying pathway in the intergenerational transmission of obesity and in adult metabolic syndrome.

There are considerable variations in the degree of mediation across the subgroups of smoking status for several CpG sites. The mediation effect appears to be stronger in smoking mother–child pairs for the following CpGs: cg04347874 (annotated gene: *NKX2-1*), cg09545197 (*KCNA3*), cg08391482 (*SERBP1*), cg02059896 (nearest gene: *PIKFYVE*), and cg15059222 (*CAMK2B*) than in the non-smoking mother–child pairs. The combination of smoking and OWO aggravates the adverse effect on plasma cholesterol levels [65]. As animal studies have indicated, maternally driven cholesterol is an important source of cholesterol for the fetus in the first trimester of pregnancy [66]. Fetus cholesterol disturbance-induced DNAm alterations are possibly augmented. Therefore, our study suggested that maternal obesity and smoking may both be involved in the same pathway that causes offspring obesity through maternal cholesterol disturbances. Similarly, maternal overweight and smoking have been found to have a synergistic adverse effect on the development of congenital heart anomalies in offspring [67]. We furthermore suggest that future studies should include interaction calculations when exploring maternal-BMI altered DNAm.

Our study had several strengths. The longitudinal follow-up in children from birth to adolescence and the usage of the latest DNA methylation array that provides comprehensive genome-wide coverage and captures additional enhancer and intergenic regions of the genome allowed us to make insight into possible unrevealed biological pathways of transgenerational amplification of obesity. The BBC, a prospective study with long-term postnatal follow-up (from birth to adolescence), is mainly an under-represented, urban, low-income Black population. Our EWAS findings were based on the largest sample size among a racial minority population. Findings from this study may directly relate to understanding and preventing obesity and reducing health disparities in people of underrepresented race in research and have the highest rates of obesity. In addition, the interplay of maternal smoking and pre-pregnancy BMI in impacting fetal DNAm could be leveraged to offer new insight to identify maternal BMI-associated methylation sites in future studies. Such approaches are infrequently studied beyond locus-specific interactions due to computational burden [68].

Our study also had some limitations. First, residual confounding due to mother’s nutritional information, substance abuse, exposure to air pollution, children’s nutrition, and physical activity during follow-up is possible, although we have made a comprehensive adjustment for preselected potential confounders, including cell type proportion (primary confounders in the previous study [13]) and also used the recommended SVA method to remove the unknown confounding effect. The genomic control factor was only 1.01 for analysis without considering smoking interaction and 1.19 for integrating potential interaction effect. It indicated small residual confoundings or polygenic effect. The elevated tail of the QQ-plot also suggested enrichment of true signals among the top CpGs. Second, maternal pre-pregnancy BMI was calculated based on self-reported data by peripartum/postpartum interviews and may be less reliable than such information obtained pre-pregnancy or in early pregnancy. However, a previous study based on the BBC mothers observed self-reporting pre-pregnancy BMI at peripartum/postpartum interviews was highly correlated with the first trimester BMI obtained from the obstetric clinic (Pearson correlation coefficient = 0.87) [69], indicating the accuracy of reported BMI. Third, both a strength and a limitation of the BBC is that it is conducted in a population of underrepresented race; while findings from the BBC may not be generalizable to other populations, the findings apply to those most impacted by obesity and its long-term consequences. Fourthly, the observational studies are not strong enough to yield standalone causal inference compared to randomized controlled trials. Future studies are warranted to validate our findings and unravel the underlying mechanisms of identified CpGs. Fifthly, DNAm data was available only at birth. Future studies with multiple time points from birth to adolescence are expected to provide insights into the role of dynamic changes in methylation and expression levels in the progress of obesity. Future studies are also expected to expand our research and integrate critical contributors to childhood obesity, including genome, epigenome, and metabolome profile. Lastly, emerging evidence suggested the same topologically associating domains (TADs) demonstrated similar gene expression and epigenetic modification profiles [70]. The DNAm itself is also suggested to have impact on the different layers of chromatin organization [71]. Integrating the effect of TADs and DNAm patterns in the same population is promising to unravel the complex associations.

## Conclusions

We presented novel findings on associations of maternal pre-pregnancy BMI with altered DNAm at 481 CpGs in newborn cord blood and the link of altered DNAm with future development of OWO among this predominantly urban, low-income racialized minority population. Our findings highlight the importance of promoting healthy weight and smoking cessation among women who wish to become pregnant. Our findings lent further support for the potential role of epigenetic modification in the intergenerational OWO. Our findings, if futher confirmed, may also help identify theraputic targets for the early-onset obesity. Future studies are warranted to validate and elucidate the functional mechanisms of our findings.

### Supplementary Information

**Additional file 1:** **Table S1.** [Characteristics of mother-newborn pairs included versus excluded from this current study]. **Table S3.** [Gene enrichment analysis of 481 probes]. **Table S4.** [Association of maternal BMI-associated CpG sites with newborn birthweight]. **Table S5.** [Association of maternal BMI-associated CpG sites with child OWO at age 1-18 years]. **Table S6.** [The annotated gene of the identified CpGs with significant mediation effect].

**Additional file 2:** **Fig. S1.** [Distributions of DNA methylation at 8 maternal-BMI associated CpG sites in cord blood]. **Fig. S2.** [Number of CpG sites and single age-window child OWO with significant mediation effect].

**Additional file 3:** **Table S2.** 481 CpG sites significantly associated with maternal pre-pregnancy body mass index or the interplay of maternal pre-pregnancy body mass index and smoking.

## Data Availability

The data underlying this article will be available on reasonable request to the corresponding author and conditional on Institutional Review Board approval.

## Abbreviations

ACME: Average causal mediation effect
BBC: Boston Birth Cohort
BMC: Boston Medical Center
BMI: Body mass index
CpG sites: Cytosine-phosphodiester bond-guanine (CpG) dinucleotides
DNA: Deoxyribonucleic acid
DNAm: DNA methylation
EMR: Electronic medical record
EWAS: Epigenome-wide association study
FDR: False discovery rate
L&D: Labor and delivery
MDS: Multidimensional scaling
OWO: Overweight or obesity
SD: Standard deviation
SE: Standard error
SNP: Single nucleotide polymorphism
SV: Surrogate variable
