# Supplementary material for: Maternal pre-pregnancy BMI, offspring epigenome-wide DNA methylation, and childhood obesity: findings from the Boston Birth Cohort
Source: BMC Med. 2023 Aug 23;21:317. doi: 10.1186/s12916-023-03003-5 (PMC10463574; doi:10.1186/s12916-023-03003-5)
Supplement: Supplementary file 1 — Additional file 1: Table S1. [Characteristics of mother-newborn pairs included versus excluded from this current study]. Table S3. [Gene enrichment analysis of 481 probes]. Table S4. [Association of maternal BMI-associated CpG sites with newborn birthweight]. Table S5. [Association of maternal BMI-associated CpG sites with child OWO at age 1-18 years]. Table S6. [The annotated gene of the identified CpGs with significant mediation effect]. [file 12916_2023_3003_MOESM1_ESM.docx]

|  | Included (n=903) | Excluded (n=7,720) | P |
| --- | --- | --- | --- |
| **Continuous Variables (mean (SD))** |  |  |  |
| Maternal age (years) | 28.28 (6.59) | 28.17 (6.44) | 0.635 |
| Maternal pre-pregnancy BMI | 26.95 (6.46) | 25.88 (6.23) | <0.001 |
| Gestational age (weeks) | 38.62 (2.47) | 37.84 (3.29) | <0.001 |
| Birthweight (grams) | 3128 (666) | 2932 (782) | <0.001 |
| **Categorical Variables, n(%)** |  |  |  |
| Parity (>= 1 live birth) | 500 (55.4) | 4394 (56.9) | 0.277 |
| Alcohol drinking during pregnancy | 75 (8.3) | 670 (8.7) | 0.670 |
| Ever smoking during pregnancy | 164 (18.2) | 1487 (19.3) | 0.350 |
| Maternal education level (>high school) | 304 (33.7) | 2684 (34.8) | 0.305 |
| Maternal race (Black) | 641 (71.0) | 3446 (44.6) | <0.001 |
| Child sex (male) | 478 (52.9) | 3818 (49.5) | 0.048 |

# Additional file 1: Table S1. Characteristics of mother-newborn pairs included versus excluded from this current study.

# Additional file 1: Table S3. Gene enrichment analysis of 481 probes which was significantly associated with maternal prepregnancy body mass index.

| Category | Term | Count | % | P value | Genes | Fold Enrichment | Bonferroni | FDR |
| --- | --- | --- | --- | --- | --- | --- | --- | --- |
| GAD_DISEASE | Triglycerides | 24 | 7.08 | 3.56E-06 | AVEN, USP24, HDAC4, PACRG, PLEKHG1, NOTCH4, ITPK1, PPM1H, SNX10, SYT9, SOBP, PLA2G6, TMC5, LPP, QKI, FRMD5, ACOXL, IGFBPL1, ZC3H7A, PRR16, NKX2-1, SRGAP3, DIP2C, CSMD1 | 3.07 | 2.28E-03 | 2.28E-03 |
| GAD_DISEASE | Hematocrit | 12 | 3.54 | 7.78E-06 | PACRG, ACOXL, XPO4, PALLD, CCDC85A, PHACTR2, SNX10, INHBA, SERGEF, PPEF2, CSMD1, QKI | 5.70 | 4.97E-03 | 2.49E-03 |
| GAD_DISEASE | Tobacco Use Disorder | 84 | 24.78 | 1.02E-04 | AVEN, PRR4, TENM2, DOCK9, TRRAP, ZFAND6, NPY2R, MSI2, SOBP, ADARB2, BICD1, HERC4, LASP1, PHACTR2, CEP170, MALRD1, DIP2C, SPG11, PLEKHG1, PACRG, CERS6, KSR1, ITPK1, CACNA2D4, NAV2, ATP11A, TMC5, RABGGTB, EPN2, VWA8, INPP4B, LATS2, DAAM1, DAD1, PALLD, HECW2, PRR16, RIN3, PKHD1L1, ERGIC1, RAI14, CAMK2B, VAC14, USP15, SHC1, PLD5, PPM1H, CACNA1A, AKAP7, PLA2G6, SDAD1, LPP, THSD4, PRH1, PTDSS1, CCDC85A, PTK2B, CAMTA1, SRGAP3, CSMD2, BID, CPA6, CSMD1, SPTBN1, OPCML, PTPRN2, SMAD3, AUTS2, XRCC5, OSBPL3, BTBD11, ATP2B2, INHBA, SYT9, TRERF1, PTPN14, USH2A, CPPED1, TRIT1, PARP11, FA2H, CACHD1, C8ORF76, ZNF410 | 1.46 | 6.34E-02 | 2.18E-02 |

GAD_DISEASE = The Genetic Association Database.

Annotation clusters with FDR p-value < 0.05 were reported. Table contains category, enrichment term, the number and proportion of genes associated with the term, p-value for that term, the genes in the enrichment term, fold enrichment, the Bonferroni-adjusted p value and the Benjamini-Hochberf FDR p-value.

# Additional file 1: Table S4. Association of maternal body mass index-associated CpG sites with newborn birthweight.

|  | Beta (assocation with the outcome) | P value (assocation with the outcome) | P value for mediation effects | FDR for mediation effect |
| --- | --- | --- | --- | --- |
| cg04107082 | -92.51 | 0.031 | 0.040 | 0.094 |
| cg02852739 | -77.37 | 0.009 | 0.012 | 0.091 |
| cg17512748 | -48.78 | 0.017 | 0.020 | 0.091 |
| cg00715197 | -40.15 | 0.017 | 0.018 | 0.091 |
| cg20239099 | -37.15 | 0.025 | 0.032 | 0.091 |
| cg00964908 | -32.66 | 0.030 | 0.029 | 0.091 |
| cg19403610 | -31.77 | 0.028 | 0.208 | 0.258 |
| cg05280508 | -26.67 | 0.023 | 0.105 | 0.171 |
| cg08331040 | -23.79 | 0.018 | 0.256 | 0.277 |
| cg10179199 | -22.96 | 0.035 | 0.070 | 0.140 |
| cg20329028 | -18.32 | 0.037 | 0.195 | 0.258 |
| cg01371922 | -11.49 | 0.048 | 0.089 | 0.159 |
| cg21672572 | 8.17 | 0.044 | 0.138 | 0.199 |
| cg10795579 | 8.22 | 0.044 | 0.136 | 0.199 |
| cg05000824 | 10.86 | 0.047 | 0.283 | 0.295 |
| cg00415263 | 21.84 | 0.024 | 0.039 | 0.094 |
| cg26696538 | 28.12 | 0.027 | 0.031 | 0.091 |
| cg14729691 | 31.10 | 0.015 | 0.064 | 0.138 |
| cg04293218 | 36.10 | 0.001 | 0.010 | 0.091 |
| cg12033619 | 38.89 | 0.017 | 0.248 | 0.277 |
| cg01964282 | 39.65 | 0.023 | 0.025 | 0.091 |
| cg22019308 | 48.79 | 0.008 | 0.204 | 0.258 |
| cg22732635 | 55.05 | 0.008 | 0.842 | 0.842 |
| cg13656778 | 55.45 | 0.046 | 0.092 | 0.159 |
| cg18116450 | 56.92 | 0.015 | 0.224 | 0.265 |
| cg26779585 | 64.91 | 0.002 | 0.003 | 0.073 |

All models adjusted for maternal age, education level, race, parity, smoking, alcohol consumption, gestational age, child sex, cord blood cell compositions (CD8, CD4, NK, B cell, monocytes, granulocytes, nucleated red blood cells), and all surrogate variables. We performed the analyses for 481 maternal body mass index-associated CpG sites. Only significant results were reported (raw P value <0.05).

# Additional file 1: Table S5. Association of maternal body mass index-associated CpG sites with child obesity or overweight (OWO) at age 1-18 years.

|  | Beta (assocation with the outcome) | OR (assocation with the outcome) | P value (assocation with the outcome) | Mediation effect | | Outcome |
| --- | --- | --- | --- | --- | --- | --- |
|  |  |  |  | P value for mediation effect | FDR for mediation effect |  |
| cg12606400 | -0.42 | 0.66 | 0.002 | 0.126 | 0.164 | owo_1y |
| cg02334771 | 0.41 | 1.51 | 0.003 | 0.043 | 0.107 | owo_1y |
| cg20001829 | -0.48 | 0.62 | 0.005 | 0.004 | 0.080 | owo_1y |
| cg03113824 | -0.42 | 0.66 | 0.005 | 0.008 | 0.080 | owo_1y |
| cg11100113 | 0.30 | 1.35 | 0.006 | 0.125 | 0.164 | owo_1y |
| cg02024855 | 0.36 | 1.43 | 0.009 | 0.009 | 0.080 | owo_1y |
| cg07734971 | -0.33 | 0.72 | 0.010 | 0.105 | 0.151 | owo_1y |
| cg02779230 | 0.27 | 1.31 | 0.010 | 0.056 | 0.113 | owo_1y |
| cg01764370 | 0.41 | 1.51 | 0.011 | 0.608 | 0.633 | owo_1y |
| cg16969189 | 0.14 | 1.15 | 0.013 | 0.088 | 0.134 | owo_1y |
| cg23205479 | -0.18 | 0.84 | 0.020 | 0.021 | 0.107 | owo_1y |
| cg23061549 | 0.31 | 1.36 | 0.021 | 0.027 | 0.107 | owo_1y |
| cg25631876 | -0.29 | 0.75 | 0.021 | 0.648 | 0.648 | owo_1y |
| cg20975802 | -0.34 | 0.71 | 0.023 | 0.582 | 0.631 | owo_1y |
| cg16184930 | -0.27 | 0.76 | 0.026 | 0.304 | 0.344 | owo_1y |
| cg02039485 | -0.16 | 0.85 | 0.029 | 0.034 | 0.107 | owo_1y |
| cg11965976 | 0.29 | 1.33 | 0.034 | 0.209 | 0.259 | owo_1y |
| cg21046843 | -0.39 | 0.68 | 0.034 | 0.035 | 0.107 | owo_1y |
| cg06768955 | -0.06 | 0.94 | 0.037 | 0.052 | 0.112 | owo_1y |
| cg05724097 | 0.21 | 1.24 | 0.039 | 0.041 | 0.107 | owo_1y |
| cg04646928 | -0.11 | 0.90 | 0.039 | 0.077 | 0.125 | owo_1y |
| cg03222326 | 0.37 | 1.45 | 0.040 | 0.228 | 0.270 | owo_1y |
| cg04035218 | -0.19 | 0.83 | 0.041 | 0.045 | 0.107 | owo_1y |
| cg19860586 | 0.29 | 1.34 | 0.041 | 0.074 | 0.125 | owo_1y |
| cg18579327 | -0.28 | 0.76 | 0.048 | 0.044 | 0.107 | owo_1y |
| cg25023616 | -0.19 | 0.83 | 0.049 | 0.072 | 0.125 | owo_1y |
| cg00045227 | 0.29 | 1.33 | 0.015 | 0.014 | 0.070 | owo_2y |
| cg02024855 | 0.36 | 1.44 | 0.007 | 0.011 | 0.070 | owo_2y |
| cg03031823 | -0.36 | 0.69 | 0.008 | 0.062 | 0.113 | owo_2y |
| cg06897650 | 0.43 | 1.53 | 0.005 | 0.882 | 0.882 | owo_2y |
| cg09023264 | 0.35 | 1.42 | 0.005 | 0.339 | 0.452 | owo_2y |
| cg10591926 | 0.43 | 1.54 | 0.001 | 0.001 | 0.016 | owo_2y |
| cg11179184 | -0.06 | 0.94 | 0.024 | 0.033 | 0.082 | owo_2y |
| cg11774251 | 0.34 | 1.41 | 0.003 | 0.011 | 0.070 | owo_2y |
| cg12033619 | 0.28 | 1.33 | 0.031 | 0.384 | 0.480 | owo_2y |
| cg15213050 | 0.40 | 1.49 | 0.003 | 0.026 | 0.074 | owo_2y |
| cg15703221 | 0.31 | 1.37 | 0.006 | 0.045 | 0.090 | owo_2y |
| cg20523008 | 0.39 | 1.47 | 0.022 | 0.072 | 0.119 | owo_2y |
| cg22139025 | -0.29 | 0.75 | 0.033 | 0.025 | 0.074 | owo_2y |
| cg23578243 | 0.25 | 1.28 | 0.024 | 0.085 | 0.131 | owo_2y |
| cg23732384 | 0.36 | 1.43 | 0.045 | 0.040 | 0.089 | owo_2y |
| cg24005667 | 0.24 | 1.28 | 0.031 | 0.875 | 0.882 | owo_2y |
| cg24540678 | -0.25 | 0.78 | 0.022 | 0.162 | 0.232 | owo_2y |
| cg25488288 | 0.24 | 1.28 | 0.026 | 0.026 | 0.074 | owo_2y |
| cg25631876 | -0.18 | 0.83 | 0.007 | 0.606 | 0.673 | owo_2y |
| cg26968269 | -0.35 | 0.71 | 0.007 | 0.482 | 0.567 | owo_2y |
| cg02059896 | -0.44 | 0.65 | 0.000 | 0.000 | 0.011 | owo_3y |
| cg14257335 | -0.27 | 0.77 | 0.003 | 0.004 | 0.041 | owo_3y |
| cg15059222 | -0.49 | 0.61 | 0.002 | 0.003 | 0.041 | owo_3y |
| cg27662877 | 0.26 | 1.30 | 0.006 | 0.007 | 0.050 | owo_3y |
| cg01886035 | -0.19 | 0.83 | 0.035 | 0.036 | 0.062 | owo_3y |
| cg02284155 | 0.31 | 1.36 | 0.018 | 0.024 | 0.062 | owo_3y |
| cg03031823 | -0.38 | 0.68 | 0.002 | 0.044 | 0.062 | owo_3y |
| cg06292624 | 0.27 | 1.31 | 0.041 | 0.040 | 0.062 | owo_3y |
| cg06599514 | 0.32 | 1.37 | 0.029 | 0.032 | 0.062 | owo_3y |
| cg07988695 | -0.34 | 0.71 | 0.023 | 0.039 | 0.062 | owo_3y |
| cg11774251 | 0.30 | 1.35 | 0.021 | 0.030 | 0.062 | owo_3y |
| cg13331383 | 0.21 | 1.24 | 0.020 | 0.022 | 0.062 | owo_3y |
| cg15969276 | 0.10 | 1.10 | 0.037 | 0.046 | 0.062 | owo_3y |
| cg17076222 | 0.14 | 1.15 | 0.049 | 0.044 | 0.062 | owo_3y |
| cg18911300 | 0.23 | 1.26 | 0.024 | 0.047 | 0.062 | owo_3y |
| cg20001829 | -0.13 | 0.88 | 0.031 | 0.026 | 0.062 | owo_3y |
| cg22139025 | -0.25 | 0.78 | 0.033 | 0.038 | 0.062 | owo_3y |
| cg22645159 | -0.12 | 0.89 | 0.029 | 0.039 | 0.062 | owo_3y |
| cg25023616 | -0.21 | 0.81 | 0.010 | 0.038 | 0.062 | owo_3y |
| cg25423135 | -0.25 | 0.78 | 0.041 | 0.040 | 0.062 | owo_3y |
| cg25488288 | 0.22 | 1.24 | 0.036 | 0.035 | 0.062 | owo_3y |
| cg08469255 | 0.52 | 1.68 | 0.046 | 0.054 | 0.066 | owo_3y |
| cg15703221 | 0.33 | 1.39 | 0.003 | 0.052 | 0.066 | owo_3y |
| cg03982537 | 0.32 | 1.37 | 0.039 | 0.096 | 0.112 | owo_3y |
| cg07686439 | -0.55 | 0.58 | 0.005 | 0.356 | 0.399 | owo_3y |
| cg26968269 | -0.35 | 0.70 | 0.002 | 0.476 | 0.512 | owo_3y |
| cg06897650 | 0.18 | 1.19 | 0.025 | 0.903 | 0.903 | owo_3y |
| cg24005667 | 0.19 | 1.21 | 0.028 | 0.895 | 0.903 | owo_3y |
| cg01023755 | 0.10 | 1.11 | 0.016 | 0.059 | 0.088 | owo_4y |
| cg03031823 | -0.31 | 0.73 | 0.010 | 0.058 | 0.088 | owo_4y |
| cg03982537 | 0.30 | 1.35 | 0.031 | 0.080 | 0.113 | owo_4y |
| cg04383707 | 0.38 | 1.46 | 0.003 | 0.004 | 0.085 | owo_4y |
| cg05376919 | 0.23 | 1.26 | 0.040 | 0.050 | 0.085 | owo_4y |
| cg06292624 | 0.24 | 1.27 | 0.021 | 0.026 | 0.085 | owo_4y |
| cg06897650 | 0.14 | 1.15 | 0.046 | 0.907 | 0.907 | owo_4y |
| cg08469255 | 0.62 | 1.87 | 0.015 | 0.016 | 0.085 | owo_4y |
| cg08937613 | 0.16 | 1.18 | 0.029 | 0.247 | 0.296 | owo_4y |
| cg10483909 | -0.20 | 0.81 | 0.027 | 0.129 | 0.163 | owo_4y |
| cg11339707 | -0.25 | 0.78 | 0.011 | 0.101 | 0.134 | owo_4y |
| cg11518020 | -0.19 | 0.82 | 0.026 | 0.043 | 0.085 | owo_4y |
| cg11774251 | 0.31 | 1.36 | 0.019 | 0.024 | 0.085 | owo_4y |
| cg13331383 | 0.19 | 1.20 | 0.038 | 0.040 | 0.085 | owo_4y |
| cg14257335 | -0.19 | 0.83 | 0.037 | 0.030 | 0.085 | owo_4y |
| cg16594299 | 0.23 | 1.25 | 0.024 | 0.026 | 0.085 | owo_4y |
| cg16814689 | 0.20 | 1.22 | 0.033 | 0.037 | 0.085 | owo_4y |
| cg18282453 | -0.37 | 0.69 | 0.047 | 0.319 | 0.364 | owo_4y |
| cg21444593 | -0.19 | 0.83 | 0.041 | 0.040 | 0.085 | owo_4y |
| cg21610602 | 0.21 | 1.23 | 0.036 | 0.047 | 0.085 | owo_4y |
| cg22139025 | -0.19 | 0.83 | 0.043 | 0.046 | 0.085 | owo_4y |
| cg23967894 | -0.18 | 0.84 | 0.041 | 0.443 | 0.483 | owo_4y |
| cg25023616 | -0.27 | 0.76 | 0.006 | 0.036 | 0.085 | owo_4y |
| cg26968269 | -0.38 | 0.68 | 0.001 | 0.490 | 0.511 | owo_4y |
| cg00486259 | 0.20 | 1.22 | 0.018 | 0.022 | 0.095 | owo_5y |
| cg04383707 | 0.32 | 1.38 | 0.011 | 0.011 | 0.095 | owo_5y |
| cg04390217 | -0.20 | 0.82 | 0.032 | 0.032 | 0.095 | owo_5y |
| cg06292624 | 0.24 | 1.27 | 0.034 | 0.038 | 0.095 | owo_5y |
| cg06897650 | 0.07 | 1.07 | 0.043 | 0.917 | 0.917 | owo_5y |
| cg07988695 | -0.27 | 0.77 | 0.017 | 0.028 | 0.095 | owo_5y |
| cg08469255 | 0.57 | 1.76 | 0.047 | 0.050 | 0.095 | owo_5y |
| cg08937613 | 0.19 | 1.21 | 0.037 | 0.252 | 0.300 | owo_5y |
| cg11100113 | 0.20 | 1.23 | 0.039 | 0.151 | 0.189 | owo_5y |
| cg11339707 | -0.25 | 0.78 | 0.007 | 0.081 | 0.106 | owo_5y |
| cg11518020 | -0.14 | 0.87 | 0.047 | 0.070 | 0.106 | owo_5y |
| cg11774251 | 0.29 | 1.34 | 0.032 | 0.046 | 0.095 | owo_5y |
| cg13331383 | 0.29 | 1.34 | 0.001 | 0.000 | 0.010 | owo_5y |
| cg14257335 | -0.19 | 0.83 | 0.038 | 0.036 | 0.095 | owo_5y |
| cg14293223 | 0.28 | 1.32 | 0.006 | 0.623 | 0.677 | owo_5y |
| cg14424414 | 0.19 | 1.20 | 0.033 | 0.057 | 0.095 | owo_5y |
| cg15703221 | 0.19 | 1.20 | 0.035 | 0.079 | 0.106 | owo_5y |
| cg16594299 | 0.15 | 1.16 | 0.040 | 0.037 | 0.095 | owo_5y |
| cg17718679 | -0.32 | 0.73 | 0.049 | 0.055 | 0.095 | owo_5y |
| cg17886022 | -0.23 | 0.80 | 0.023 | 0.025 | 0.095 | owo_5y |
| cg18911300 | 0.20 | 1.23 | 0.038 | 0.073 | 0.106 | owo_5y |
| cg19860734 | 0.21 | 1.24 | 0.043 | 0.042 | 0.095 | owo_5y |
| cg21578090 | -0.19 | 0.82 | 0.044 | 0.481 | 0.547 | owo_5y |
| cg24005667 | 0.25 | 1.29 | 0.001 | 0.858 | 0.893 | owo_5y |
| cg26696538 | -0.16 | 0.85 | 0.033 | 0.029 | 0.095 | owo_5y |
| cg00486259 | 0.16 | 1.18 | 0.043 | 0.054 | 0.078 | owo_6y |
| cg01886035 | -0.18 | 0.84 | 0.049 | 0.051 | 0.078 | owo_6y |
| cg01964282 | 0.17 | 1.18 | 0.047 | 0.052 | 0.078 | owo_6y |
| cg02059896 | -0.31 | 0.74 | 0.015 | 0.018 | 0.078 | owo_6y |
| cg04347874 | 0.25 | 1.29 | 0.030 | 0.031 | 0.078 | owo_6y |
| cg04383707 | 0.33 | 1.39 | 0.009 | 0.013 | 0.078 | owo_6y |
| cg06292624 | 0.28 | 1.32 | 0.047 | 0.054 | 0.078 | owo_6y |
| cg06897650 | 0.19 | 1.21 | 0.006 | 0.896 | 0.896 | owo_6y |
| cg07863022 | -0.47 | 0.63 | 0.031 | 0.032 | 0.078 | owo_6y |
| cg07988695 | -0.37 | 0.69 | 0.005 | 0.018 | 0.078 | owo_6y |
| cg08391482 | 0.16 | 1.18 | 0.040 | 0.034 | 0.078 | owo_6y |
| cg09021643 | 0.13 | 1.14 | 0.030 | 0.032 | 0.078 | owo_6y |
| cg11179184 | -0.13 | 0.87 | 0.033 | 0.042 | 0.078 | owo_6y |
| cg11339707 | -0.20 | 0.82 | 0.025 | 0.106 | 0.125 | owo_6y |
| cg11774251 | 0.30 | 1.35 | 0.029 | 0.038 | 0.078 | owo_6y |
| cg11789146 | 0.16 | 1.17 | 0.023 | 0.057 | 0.078 | owo_6y |
| cg13331383 | 0.23 | 1.26 | 0.013 | 0.016 | 0.078 | owo_6y |
| cg14257335 | -0.18 | 0.84 | 0.037 | 0.035 | 0.078 | owo_6y |
| cg14412459 | -0.19 | 0.82 | 0.028 | 0.072 | 0.088 | owo_6y |
| cg17886022 | -0.26 | 0.77 | 0.006 | 0.008 | 0.078 | owo_6y |
| cg18911300 | 0.19 | 1.21 | 0.041 | 0.066 | 0.084 | owo_6y |
| cg21610602 | 0.19 | 1.21 | 0.043 | 0.058 | 0.078 | owo_6y |
| cg22139025 | -0.21 | 0.81 | 0.043 | 0.050 | 0.078 | owo_6y |
| cg24005667 | 0.27 | 1.31 | 0.001 | 0.874 | 0.896 | owo_6y |
| cg26696538 | -0.15 | 0.86 | 0.044 | 0.048 | 0.078 | owo_6y |
| cg26968269 | -0.30 | 0.74 | 0.012 | 0.486 | 0.524 | owo_6y |
| cg27227159 | 0.22 | 1.25 | 0.048 | 0.238 | 0.268 | owo_6y |
| cg01886035 | -0.17 | 0.84 | 0.046 | 0.046 | 0.069 | owo_7y |
| cg02059896 | -0.29 | 0.75 | 0.031 | 0.030 | 0.067 | owo_7y |
| cg04294170 | 0.19 | 1.21 | 0.048 | 0.830 | 0.907 | owo_7y |
| cg04347874 | 0.29 | 1.34 | 0.005 | 0.005 | 0.043 | owo_7y |
| cg04383707 | 0.26 | 1.29 | 0.045 | 0.050 | 0.069 | owo_7y |
| cg06292624 | 0.32 | 1.38 | 0.002 | 0.007 | 0.043 | owo_7y |
| cg06897650 | 0.20 | 1.22 | 0.025 | 0.907 | 0.907 | owo_7y |
| cg08937613 | 0.18 | 1.20 | 0.037 | 0.242 | 0.290 | owo_7y |
| cg09545197 | 0.32 | 1.38 | 0.001 | 0.007 | 0.043 | owo_7y |
| cg09949429 | 0.03 | 1.03 | 0.045 | 0.041 | 0.069 | owo_7y |
| cg12152256 | 0.18 | 1.20 | 0.033 | 0.041 | 0.069 | owo_7y |
| cg13331383 | 0.23 | 1.25 | 0.014 | 0.016 | 0.049 | owo_7y |
| cg14015044 | 0.26 | 1.30 | 0.027 | 0.028 | 0.067 | owo_7y |
| cg17886022 | -0.28 | 0.75 | 0.009 | 0.010 | 0.043 | owo_7y |
| cg21610602 | 0.13 | 1.14 | 0.034 | 0.048 | 0.069 | owo_7y |
| cg23732384 | 0.20 | 1.22 | 0.017 | 0.016 | 0.049 | owo_7y |
| cg24005667 | 0.21 | 1.23 | 0.004 | 0.870 | 0.907 | owo_7y |
| cg26831560 | -0.29 | 0.75 | 0.013 | 0.079 | 0.102 | owo_7y |
| cg01241697 | 0.18 | 1.20 | 0.029 | 0.035 | 0.066 | owo_8y |
| cg04036537 | 0.23 | 1.26 | 0.041 | 0.051 | 0.077 | owo_8y |
| cg04347874 | 0.23 | 1.26 | 0.007 | 0.011 | 0.045 | owo_8y |
| cg04390217 | -0.21 | 0.81 | 0.014 | 0.013 | 0.045 | owo_8y |
| cg05000824 | 0.21 | 1.24 | 0.034 | 0.340 | 0.376 | owo_8y |
| cg06292624 | 0.34 | 1.40 | 0.001 | 0.004 | 0.045 | owo_8y |
| cg08391482 | 0.17 | 1.19 | 0.021 | 0.021 | 0.055 | owo_8y |
| cg08937613 | 0.17 | 1.19 | 0.039 | 0.245 | 0.286 | owo_8y |
| cg09545197 | 0.25 | 1.29 | 0.006 | 0.012 | 0.045 | owo_8y |
| cg11100113 | 0.16 | 1.17 | 0.027 | 0.149 | 0.196 | owo_8y |
| cg11339707 | -0.20 | 0.82 | 0.044 | 0.120 | 0.167 | owo_8y |
| cg13331383 | 0.21 | 1.23 | 0.033 | 0.033 | 0.066 | owo_8y |
| cg14015044 | 0.31 | 1.37 | 0.010 | 0.010 | 0.045 | owo_8y |
| cg14257335 | -0.19 | 0.83 | 0.046 | 0.047 | 0.076 | owo_8y |
| cg17886022 | -0.32 | 0.72 | 0.006 | 0.005 | 0.045 | owo_8y |
| cg19166733 | 0.24 | 1.27 | 0.048 | 0.182 | 0.224 | owo_8y |
| cg21610602 | 0.20 | 1.22 | 0.022 | 0.032 | 0.066 | owo_8y |
| cg23560320 | 0.13 | 1.14 | 0.038 | 0.524 | 0.550 | owo_8y |
| cg24005667 | 0.25 | 1.28 | 0.001 | 0.878 | 0.878 | owo_8y |
| cg25378362 | -0.14 | 0.87 | 0.018 | 0.017 | 0.050 | owo_8y |
| cg27236173 | -0.06 | 0.95 | 0.043 | 0.045 | 0.076 | owo_8y |
| cg00486259 | 0.11 | 1.11 | 0.044 | 0.043 | 0.065 | owo_9y |
| cg00715197 | 0.21 | 1.24 | 0.022 | 0.020 | 0.055 | owo_9y |
| cg01241697 | 0.21 | 1.23 | 0.023 | 0.029 | 0.056 | owo_9y |
| cg01886035 | -0.23 | 0.79 | 0.012 | 0.009 | 0.050 | owo_9y |
| cg02779230 | -0.22 | 0.80 | 0.046 | 0.098 | 0.133 | owo_9y |
| cg03046233 | -0.19 | 0.82 | 0.038 | 0.045 | 0.065 | owo_9y |
| cg04347874 | 0.29 | 1.34 | 0.004 | 0.005 | 0.050 | owo_9y |
| cg04383707 | 0.26 | 1.30 | 0.036 | 0.032 | 0.057 | owo_9y |
| cg04390217 | -0.22 | 0.80 | 0.014 | 0.011 | 0.050 | owo_9y |
| cg05000824 | 0.23 | 1.26 | 0.021 | 0.352 | 0.386 | owo_9y |
| cg06292624 | 0.38 | 1.46 | 0.004 | 0.007 | 0.050 | owo_9y |
| cg07988695 | -0.39 | 0.67 | 0.008 | 0.016 | 0.054 | owo_9y |
| cg08937613 | 0.20 | 1.22 | 0.020 | 0.241 | 0.277 | owo_9y |
| cg09545197 | 0.25 | 1.28 | 0.006 | 0.016 | 0.054 | owo_9y |
| cg11100113 | 0.16 | 1.17 | 0.031 | 0.142 | 0.172 | owo_9y |
| cg14015044 | 0.26 | 1.30 | 0.022 | 0.022 | 0.055 | owo_9y |
| cg15599946 | -0.20 | 0.82 | 0.038 | 0.111 | 0.142 | owo_9y |
| cg16594299 | 0.14 | 1.14 | 0.037 | 0.028 | 0.056 | owo_9y |
| cg17886022 | -0.33 | 0.72 | 0.003 | 0.002 | 0.046 | owo_9y |
| cg21610602 | 0.20 | 1.22 | 0.016 | 0.027 | 0.056 | owo_9y |
| cg23560320 | 0.21 | 1.23 | 0.012 | 0.474 | 0.496 | owo_9y |
| cg23732384 | 0.14 | 1.15 | 0.036 | 0.036 | 0.058 | owo_9y |
| cg24005667 | 0.19 | 1.20 | 0.002 | 0.884 | 0.884 | owo_9y |
| cg00625573 | -0.12 | 0.88 | 0.045 | 0.048 | 0.071 | owo_10y |
| cg04347874 | 0.24 | 1.27 | 0.013 | 0.014 | 0.064 | owo_10y |
| cg04383707 | 0.29 | 1.34 | 0.021 | 0.029 | 0.064 | owo_10y |
| cg04390217 | -0.26 | 0.77 | 0.009 | 0.009 | 0.064 | owo_10y |
| cg05000824 | 0.26 | 1.30 | 0.010 | 0.352 | 0.394 | owo_10y |
| cg06292624 | 0.43 | 1.54 | 0.001 | 0.003 | 0.053 | owo_10y |
| cg08391482 | 0.18 | 1.20 | 0.031 | 0.028 | 0.064 | owo_10y |
| cg08937613 | 0.26 | 1.30 | 0.005 | 0.235 | 0.279 | owo_10y |
| cg09545197 | 0.21 | 1.23 | 0.023 | 0.026 | 0.064 | owo_10y |
| cg12357419 | -0.12 | 0.89 | 0.039 | 0.037 | 0.064 | owo_10y |
| cg14015044 | 0.25 | 1.29 | 0.036 | 0.040 | 0.064 | owo_10y |
| cg17886022 | -0.30 | 0.74 | 0.026 | 0.023 | 0.064 | owo_10y |
| cg19166733 | 0.25 | 1.28 | 0.037 | 0.164 | 0.222 | owo_10y |
| cg21444593 | -0.36 | 0.70 | 0.038 | 0.038 | 0.064 | owo_10y |
| cg21610602 | 0.21 | 1.23 | 0.016 | 0.025 | 0.064 | owo_10y |
| cg23560320 | 0.17 | 1.19 | 0.035 | 0.524 | 0.554 | owo_10y |
| cg24005667 | 0.20 | 1.23 | 0.000 | 0.879 | 0.879 | owo_10y |
| cg27227159 | 0.21 | 1.23 | 0.049 | 0.221 | 0.279 | owo_10y |
| cg27236173 | -0.10 | 0.90 | 0.037 | 0.031 | 0.064 | owo_10y |
| cg04347874 | 0.22 | 1.25 | 0.022 | 0.019 | 0.051 | owo_11y |
| cg04383707 | 0.31 | 1.36 | 0.014 | 0.013 | 0.051 | owo_11y |
| cg04390217 | -0.24 | 0.79 | 0.015 | 0.014 | 0.051 | owo_11y |
| cg05000824 | 0.25 | 1.29 | 0.020 | 0.342 | 0.395 | owo_11y |
| cg06292624 | 0.38 | 1.46 | 0.002 | 0.008 | 0.051 | owo_11y |
| cg06897650 | 0.18 | 1.20 | 0.030 | 0.913 | 0.913 | owo_11y |
| cg08391482 | 0.20 | 1.22 | 0.020 | 0.020 | 0.051 | owo_11y |
| cg08937613 | 0.21 | 1.23 | 0.032 | 0.251 | 0.314 | owo_11y |
| cg15081358 | -0.19 | 0.83 | 0.049 | 0.232 | 0.314 | owo_11y |
| cg15909016 | 0.20 | 1.22 | 0.041 | 0.041 | 0.077 | owo_11y |
| cg17886022 | -0.31 | 0.74 | 0.011 | 0.010 | 0.051 | owo_11y |
| cg19166733 | 0.25 | 1.29 | 0.033 | 0.153 | 0.230 | owo_11y |
| cg21610602 | 0.26 | 1.30 | 0.015 | 0.026 | 0.057 | owo_11y |
| cg24005667 | 0.24 | 1.27 | 0.000 | 0.890 | 0.913 | owo_11y |
| cg26831560 | -0.25 | 0.78 | 0.029 | 0.094 | 0.156 | owo_11y |
| cg00486259 | 0.13 | 1.14 | 0.046 | 0.052 | 0.099 | owo_12y |
| cg00715197 | 0.19 | 1.21 | 0.046 | 0.045 | 0.095 | owo_12y |
| cg02852739 | 0.27 | 1.31 | 0.032 | 0.035 | 0.084 | owo_12y |
| cg04347874 | 0.20 | 1.23 | 0.033 | 0.036 | 0.084 | owo_12y |
| cg04383707 | 0.32 | 1.37 | 0.012 | 0.014 | 0.071 | owo_12y |
| cg04390217 | -0.28 | 0.76 | 0.007 | 0.010 | 0.070 | owo_12y |
| cg05000824 | 0.25 | 1.28 | 0.033 | 0.356 | 0.415 | owo_12y |
| cg06292624 | 0.35 | 1.42 | 0.005 | 0.008 | 0.070 | owo_12y |
| cg06897650 | 0.24 | 1.27 | 0.007 | 0.902 | 0.902 | owo_12y |
| cg07640083 | -0.18 | 0.84 | 0.029 | 0.267 | 0.331 | owo_12y |
| cg08391482 | 0.20 | 1.22 | 0.017 | 0.022 | 0.084 | owo_12y |
| cg08937613 | 0.19 | 1.20 | 0.041 | 0.268 | 0.331 | owo_12y |
| cg14015044 | 0.27 | 1.31 | 0.030 | 0.036 | 0.084 | owo_12y |
| cg14412459 | -0.18 | 0.83 | 0.033 | 0.076 | 0.134 | owo_12y |
| cg14935574 | -0.13 | 0.88 | 0.048 | 0.233 | 0.326 | owo_12y |
| cg17886022 | -0.31 | 0.73 | 0.008 | 0.006 | 0.070 | owo_12y |
| cg19166733 | 0.29 | 1.34 | 0.016 | 0.136 | 0.205 | owo_12y |
| cg21610602 | 0.26 | 1.30 | 0.013 | 0.028 | 0.084 | owo_12y |
| cg23560320 | 0.17 | 1.19 | 0.044 | 0.524 | 0.580 | owo_12y |
| cg24005667 | 0.16 | 1.17 | 0.003 | 0.869 | 0.902 | owo_12y |
| cg26831560 | -0.24 | 0.79 | 0.037 | 0.111 | 0.180 | owo_12y |
| cg00486259 | 0.19 | 1.21 | 0.021 | 0.032 | 0.068 | owo_13y |
| cg02852739 | 0.27 | 1.31 | 0.033 | 0.030 | 0.068 | owo_13y |
| cg03453403 | 0.14 | 1.15 | 0.042 | 0.043 | 0.076 | owo_13y |
| cg04347874 | 0.23 | 1.26 | 0.015 | 0.014 | 0.050 | owo_13y |
| cg04383707 | 0.36 | 1.43 | 0.004 | 0.006 | 0.045 | owo_13y |
| cg04390217 | -0.28 | 0.76 | 0.005 | 0.006 | 0.045 | owo_13y |
| cg05000824 | 0.26 | 1.29 | 0.028 | 0.354 | 0.413 | owo_13y |
| cg06292624 | 0.30 | 1.35 | 0.007 | 0.011 | 0.045 | owo_13y |
| cg06897650 | 0.24 | 1.27 | 0.007 | 0.898 | 0.898 | owo_13y |
| cg08391482 | 0.21 | 1.23 | 0.009 | 0.010 | 0.045 | owo_13y |
| cg08937613 | 0.26 | 1.29 | 0.011 | 0.234 | 0.289 | owo_13y |
| cg11100113 | 0.12 | 1.13 | 0.029 | 0.130 | 0.196 | owo_13y |
| cg17886022 | -0.33 | 0.72 | 0.005 | 0.005 | 0.045 | owo_13y |
| cg19166733 | 0.24 | 1.28 | 0.046 | 0.154 | 0.215 | owo_13y |
| cg21610602 | 0.31 | 1.37 | 0.008 | 0.020 | 0.059 | owo_13y |
| cg23560320 | 0.17 | 1.19 | 0.033 | 0.513 | 0.567 | owo_13y |
| cg24005667 | 0.17 | 1.19 | 0.004 | 0.890 | 0.898 | owo_13y |
| cg25378362 | -0.07 | 0.93 | 0.026 | 0.025 | 0.066 | owo_13y |
| cg26831560 | -0.25 | 0.78 | 0.029 | 0.100 | 0.162 | owo_13y |
| cg27227159 | 0.23 | 1.25 | 0.036 | 0.229 | 0.289 | owo_13y |
| ch7137597056r | 0.04 | 1.04 | 0.042 | 0.041 | 0.076 | owo_13y |
| cg00625573 | -0.12 | 0.89 | 0.042 | 0.039 | 0.087 | owo_14y |
| cg04347874 | 0.27 | 1.31 | 0.006 | 0.007 | 0.083 | owo_14y |
| cg04383707 | 0.34 | 1.40 | 0.007 | 0.013 | 0.083 | owo_14y |
| cg04390217 | -0.24 | 0.79 | 0.014 | 0.011 | 0.083 | owo_14y |
| cg05000824 | 0.23 | 1.26 | 0.046 | 0.357 | 0.476 | owo_14y |
| cg06292624 | 0.29 | 1.34 | 0.019 | 0.022 | 0.083 | owo_14y |
| cg06897650 | 0.21 | 1.23 | 0.008 | 0.891 | 0.895 | owo_14y |
| cg07441462 | -0.21 | 0.81 | 0.028 | 0.838 | 0.895 | owo_14y |
| cg07640083 | -0.15 | 0.86 | 0.046 | 0.286 | 0.408 | owo_14y |
| cg08391482 | 0.19 | 1.21 | 0.023 | 0.022 | 0.083 | owo_14y |
| cg08937613 | 0.21 | 1.24 | 0.029 | 0.238 | 0.366 | owo_14y |
| cg09545197 | 0.18 | 1.20 | 0.046 | 0.050 | 0.100 | owo_14y |
| cg13249541 | -0.18 | 0.84 | 0.050 | 0.894 | 0.895 | owo_14y |
| cg14412459 | -0.18 | 0.84 | 0.039 | 0.090 | 0.149 | owo_14y |
| cg15909016 | 0.20 | 1.23 | 0.029 | 0.032 | 0.083 | owo_14y |
| cg17886022 | -0.27 | 0.76 | 0.033 | 0.033 | 0.083 | owo_14y |
| cg21610602 | 0.26 | 1.29 | 0.014 | 0.025 | 0.083 | owo_14y |
| cg23560320 | 0.15 | 1.16 | 0.049 | 0.530 | 0.663 | owo_14y |
| cg24005667 | 0.12 | 1.12 | 0.021 | 0.895 | 0.895 | owo_14y |
| cg26831560 | -0.27 | 0.76 | 0.017 | 0.084 | 0.149 | owo_14y |
| cg00486259 | 0.14 | 1.15 | 0.042 | 0.043 | 0.112 | owo_15y |
| cg00625573 | -0.09 | 0.91 | 0.045 | 0.053 | 0.113 | owo_15y |
| cg04347874 | 0.27 | 1.31 | 0.005 | 0.006 | 0.071 | owo_15y |
| cg04383707 | 0.33 | 1.40 | 0.007 | 0.007 | 0.071 | owo_15y |
| cg04390217 | -0.22 | 0.80 | 0.014 | 0.018 | 0.097 | owo_15y |
| cg06292624 | 0.24 | 1.27 | 0.026 | 0.028 | 0.097 | owo_15y |
| cg06897650 | 0.20 | 1.22 | 0.008 | 0.902 | 0.902 | owo_15y |
| cg07441462 | -0.20 | 0.82 | 0.031 | 0.825 | 0.902 | owo_15y |
| cg08391482 | 0.18 | 1.20 | 0.028 | 0.025 | 0.097 | owo_15y |
| cg08937613 | 0.20 | 1.22 | 0.038 | 0.252 | 0.353 | owo_15y |
| cg11100113 | 0.12 | 1.13 | 0.040 | 0.140 | 0.211 | owo_15y |
| cg13249541 | -0.20 | 0.82 | 0.035 | 0.887 | 0.902 | owo_15y |
| cg13571568 | 0.20 | 1.22 | 0.042 | 0.054 | 0.113 | owo_15y |
| cg14412459 | -0.18 | 0.84 | 0.038 | 0.083 | 0.145 | owo_15y |
| cg15909016 | 0.18 | 1.20 | 0.048 | 0.059 | 0.113 | owo_15y |
| cg17886022 | -0.29 | 0.75 | 0.025 | 0.033 | 0.098 | owo_15y |
| cg21610602 | 0.28 | 1.32 | 0.010 | 0.020 | 0.097 | owo_15y |
| cg23560320 | 0.17 | 1.19 | 0.032 | 0.530 | 0.696 | owo_15y |
| cg24005667 | 0.13 | 1.14 | 0.011 | 0.878 | 0.902 | owo_15y |
| cg26831560 | -0.25 | 0.78 | 0.029 | 0.105 | 0.170 | owo_15y |
| cg27386563 | -0.36 | 0.70 | 0.025 | 0.692 | 0.855 | owo_15y |
| cg00486259 | 0.15 | 1.16 | 0.039 | 0.049 | 0.098 | owo_16y |
| cg00625573 | -0.12 | 0.89 | 0.041 | 0.039 | 0.098 | owo_16y |
| cg03046233 | -0.20 | 0.82 | 0.044 | 0.043 | 0.098 | owo_16y |
| cg04347874 | 0.24 | 1.27 | 0.017 | 0.019 | 0.098 | owo_16y |
| cg04383707 | 0.33 | 1.39 | 0.008 | 0.008 | 0.098 | owo_16y |
| cg04390217 | -0.23 | 0.79 | 0.013 | 0.014 | 0.098 | owo_16y |
| cg06897650 | 0.17 | 1.18 | 0.010 | 0.907 | 0.907 | owo_16y |
| cg07441462 | -0.19 | 0.83 | 0.041 | 0.804 | 0.907 | owo_16y |
| cg08391482 | 0.17 | 1.19 | 0.034 | 0.034 | 0.098 | owo_16y |
| cg08937613 | 0.24 | 1.27 | 0.016 | 0.237 | 0.316 | owo_16y |
| cg11100113 | 0.11 | 1.12 | 0.045 | 0.157 | 0.225 | owo_16y |
| cg12533806 | 0.18 | 1.20 | 0.050 | 0.049 | 0.098 | owo_16y |
| cg13249541 | -0.21 | 0.81 | 0.029 | 0.898 | 0.907 | owo_16y |
| cg14412459 | -0.20 | 0.82 | 0.023 | 0.064 | 0.106 | owo_16y |
| cg15428792 | 0.02 | 1.02 | 0.048 | 0.061 | 0.106 | owo_16y |
| cg17886022 | -0.29 | 0.75 | 0.029 | 0.028 | 0.098 | owo_16y |
| cg21610602 | 0.24 | 1.27 | 0.013 | 0.022 | 0.098 | owo_16y |
| cg23560320 | 0.18 | 1.20 | 0.026 | 0.542 | 0.677 | owo_16y |
| cg24005667 | 0.14 | 1.15 | 0.013 | 0.885 | 0.907 | owo_16y |
| cg26831560 | -0.26 | 0.77 | 0.024 | 0.087 | 0.134 | owo_16y |
| cg00486259 | 0.15 | 1.16 | 0.039 | 0.049 | 0.102 | owo_17y |
| cg00625573 | -0.12 | 0.89 | 0.039 | 0.041 | 0.102 | owo_17y |
| cg04347874 | 0.25 | 1.29 | 0.011 | 0.012 | 0.102 | owo_17y |
| cg04383707 | 0.34 | 1.41 | 0.006 | 0.012 | 0.102 | owo_17y |
| cg04390217 | -0.22 | 0.80 | 0.015 | 0.017 | 0.102 | owo_17y |
| cg06292624 | 0.23 | 1.26 | 0.040 | 0.047 | 0.102 | owo_17y |
| cg06897650 | 0.18 | 1.20 | 0.009 | 0.896 | 0.901 | owo_17y |
| cg07441462 | -0.21 | 0.81 | 0.026 | 0.804 | 0.901 | owo_17y |
| cg08391482 | 0.18 | 1.20 | 0.028 | 0.030 | 0.102 | owo_17y |
| cg08937613 | 0.22 | 1.25 | 0.021 | 0.238 | 0.313 | owo_17y |
| cg09545197 | 0.18 | 1.19 | 0.050 | 0.063 | 0.109 | owo_17y |
| cg11100113 | 0.11 | 1.12 | 0.045 | 0.154 | 0.216 | owo_17y |
| cg12533806 | 0.18 | 1.20 | 0.047 | 0.042 | 0.102 | owo_17y |
| cg13249541 | -0.21 | 0.81 | 0.030 | 0.901 | 0.901 | owo_17y |
| cg13571568 | 0.20 | 1.22 | 0.044 | 0.060 | 0.109 | owo_17y |
| cg14412459 | -0.19 | 0.82 | 0.025 | 0.068 | 0.109 | owo_17y |
| cg17886022 | -0.29 | 0.75 | 0.027 | 0.025 | 0.102 | owo_17y |
| cg21610602 | 0.25 | 1.29 | 0.012 | 0.021 | 0.102 | owo_17y |
| cg23560320 | 0.17 | 1.19 | 0.028 | 0.502 | 0.620 | owo_17y |
| cg24005667 | 0.15 | 1.16 | 0.009 | 0.859 | 0.901 | owo_17y |
| cg26831560 | -0.25 | 0.78 | 0.029 | 0.095 | 0.142 | owo_17y |
| cg06897650 | 0.20 | 1.22 | 0.007 | 0.885 | 0.901 | owo_18y |
| cg24005667 | 0.15 | 1.16 | 0.008 | 0.864 | 0.901 | owo_18y |
| cg04347874 | 0.26 | 1.30 | 0.010 | 0.010 | 0.091 | owo_18y |
| cg21610602 | 0.26 | 1.29 | 0.012 | 0.025 | 0.091 | owo_18y |
| cg04383707 | 0.31 | 1.37 | 0.012 | 0.011 | 0.091 | owo_18y |
| cg04390217 | -0.22 | 0.80 | 0.014 | 0.018 | 0.091 | owo_18y |
| cg17886022 | -0.32 | 0.73 | 0.014 | 0.014 | 0.091 | owo_18y |
| cg08937613 | 0.24 | 1.27 | 0.016 | 0.246 | 0.318 | owo_18y |
| cg14412459 | -0.20 | 0.82 | 0.023 | 0.066 | 0.112 | owo_18y |
| cg07441462 | -0.22 | 0.80 | 0.024 | 0.823 | 0.901 | owo_18y |
| cg08391482 | 0.18 | 1.20 | 0.027 | 0.024 | 0.091 | owo_18y |
| cg13249541 | -0.20 | 0.82 | 0.031 | 0.901 | 0.901 | owo_18y |
| cg26831560 | -0.25 | 0.78 | 0.031 | 0.110 | 0.173 | owo_18y |
| cg15909016 | 0.21 | 1.23 | 0.032 | 0.037 | 0.091 | owo_18y |
| cg00625573 | -0.13 | 0.88 | 0.034 | 0.037 | 0.091 | owo_18y |
| cg25378362 | -0.09 | 0.92 | 0.035 | 0.037 | 0.091 | owo_18y |
| cg00486259 | 0.16 | 1.17 | 0.037 | 0.044 | 0.098 | owo_18y |
| cg11100113 | 0.13 | 1.14 | 0.038 | 0.163 | 0.239 | owo_18y |
| cg06292624 | 0.22 | 1.25 | 0.041 | 0.050 | 0.099 | owo_18y |
| cg13571568 | 0.20 | 1.22 | 0.046 | 0.056 | 0.103 | owo_18y |
| cg27227159 | 0.17 | 1.18 | 0.048 | 0.232 | 0.318 | owo_18y |
| cg23560320 | 0.15 | 1.16 | 0.049 | 0.521 | 0.637 | owo_18y |

All models adjusted for maternal age, education level, race, parity, smoking, alcohol consumption, gestational age, child sex, birthweight, cord blood cell compositions (CD8, CD4, NK, B cell, monocytes, granulocytes, nucleated red blood cells), and all surrogate variables. We performed the analyses for 481 maternal body mass index-associated CpG sites. Only significant results were reported (raw P value <0.05).

**Additional file 1: Table S6. The annotated or nearest annotated gene of the identified CpGs with significant mediation effect in our study and the previous GWAS findings**

| Gene | CpG | Associations reported in GWAS catalog | | | | |
| --- | --- | --- | --- | --- | --- | --- |
|  |  | Physical measurements | Adult Metabolic syndrome | Adolescent disease | Blood cell | Others |
| *AKAP7* | cg06292624 | Weight^35^; Hip circumference adjusted for BMI^36^; Height^34^ |  | Adolescent idiopathic scoliosis^37^ | Mean corpuscular hemoglobin^38^; Mean corpuscular volume^38–40^; Platelet count^38^ |  |
| *LINC00174* | cg17886022 |  |  |  |  |  |
| *NKX2-1* | cg04347874 | Height^34,35^; Hip circumference adjusted for BMI^36^ |  |  |  | Educational attainment^41^ |
| *PECR* | cg04390217 |  |  |  |  | Alcohol dependence^42,43^ |
| *KCNA3* | cg09545197 |  |  | Acute lymphoblastic leukemia (childhood)^44^ | Monocyte count or percentage^38,40^; White blood cell count^45^ |  |
| *TMEM203* | cg13331383 |  |  |  |  |  |
| *NDOR1* |  |  | Glycated hemoglobin levels^47^ |  | Mean corpuscular hemoglobin^35,40,46^; Red blood cell count^34,40^ |  |
| *NCCRP1* | cg04383707 |  |  |  |  |  |
| *SERBP1* | cg08391482 |  |  |  |  | Educational attainment^48^ |
| *CGREF1* | cg01886035 |  | Total cholesterol levels^47^; Triglyceride measurement^47^ |  |  | Gamma glutamyl transferase levels^47^ |
| *TNFRSF10C* | cg14015044 |  |  |  | Basophil count^38^; White blood cell count^38,46^; Mean corpuscular volume^46^; Neutrophil count^46^ |  |
| *MAP2K7* | cg23732384 |  | HDL cholesterol levels^49^; Type 2 diabetes^34,35,50,51^; Fasting insulin^52^ |  | Mean corpuscular hemoglobin concentration^40^ |  |
| *PIKFYVE** | cg02059896 |  |  |  |  | Adult asthma^53^ |
| *KLHL22* | cg14257335 |  |  |  |  |  |
| *CAMK2B* | cg15059222 | Birth weight^54^ | Metabolic syndrome^56^; Systolic blood pressure^57^; Type 2 diabetes^35,51^; Glycated hemoglobin levels^47^; Fasting blood glucose^47,52,58^; Two-hour glucose^52^ |  |  | Educational attainment^59^; Osteoarthritis (hip)^55^ |

*For inter-genic CpG sites, we used the UCSC Genome Browser on Human (GRCh37/hg19) to locate the nearest annotated gene.
