# Supplementary material for: Maternal pre-pregnancy BMI, offspring epigenome-wide DNA methylation, and childhood obesity: findings from the Boston Birth Cohort
Source: BMC Med. 2023 Aug 23;21:317. doi: 10.1186/s12916-023-03003-5 (PMC10463574; doi:10.1186/s12916-023-03003-5)
Supplement: Supplementary file 2 — Additional file 2: Fig. S1. [Distributions of DNA methylation at 8 maternal-BMI associated CpG sites in cord blood]. Fig. S2. [Number of CpG sites and single age-window child OWO with significant mediation effect]. [file 12916_2023_3003_MOESM2_ESM.docx]

Additional file 2: Fig. S1. Distributions of DNA methylation at 8 maternal-BMI associated CpG sites in cord blood, stratified by maternal obesity status (under/normal [U/N] weight, overweight, and obesity).


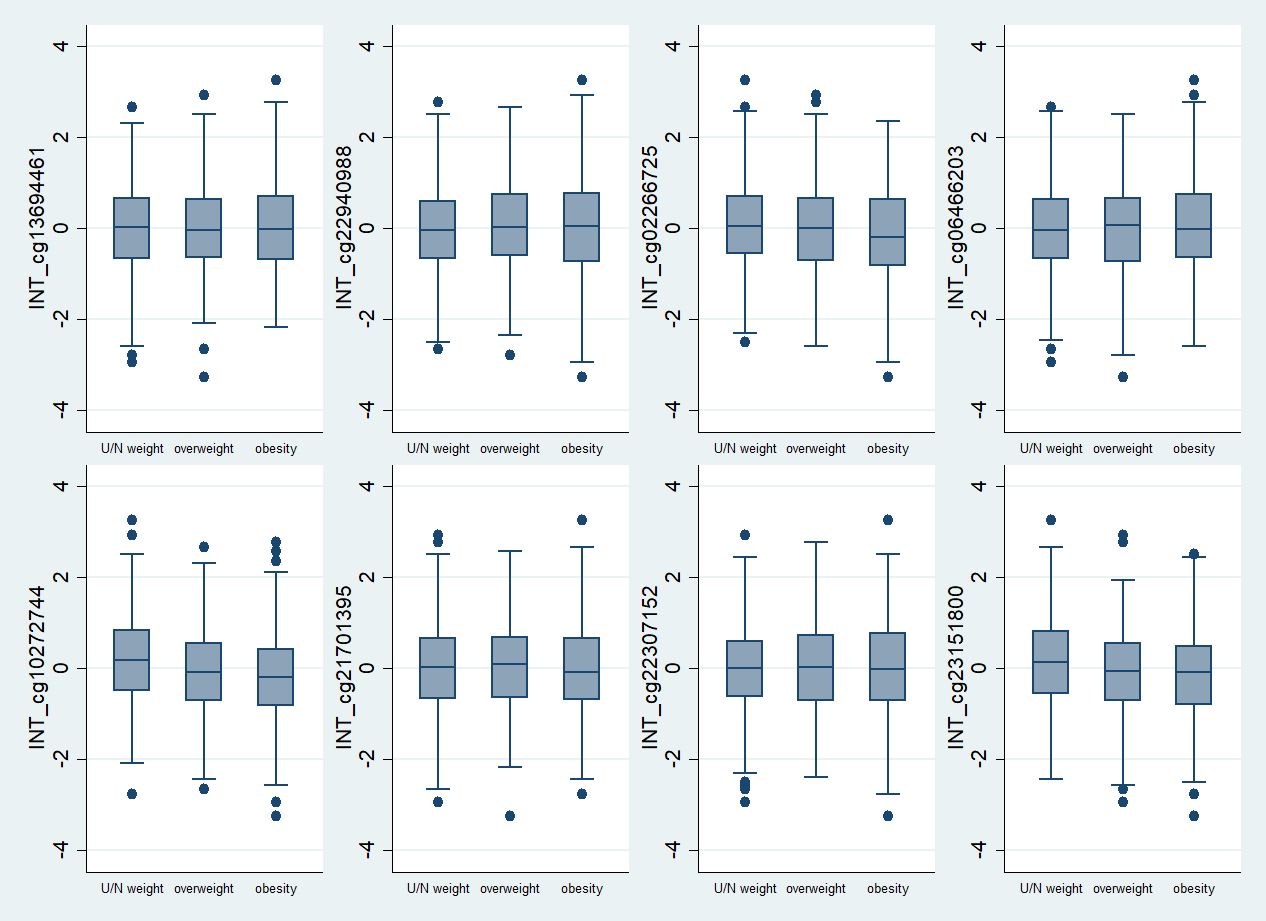


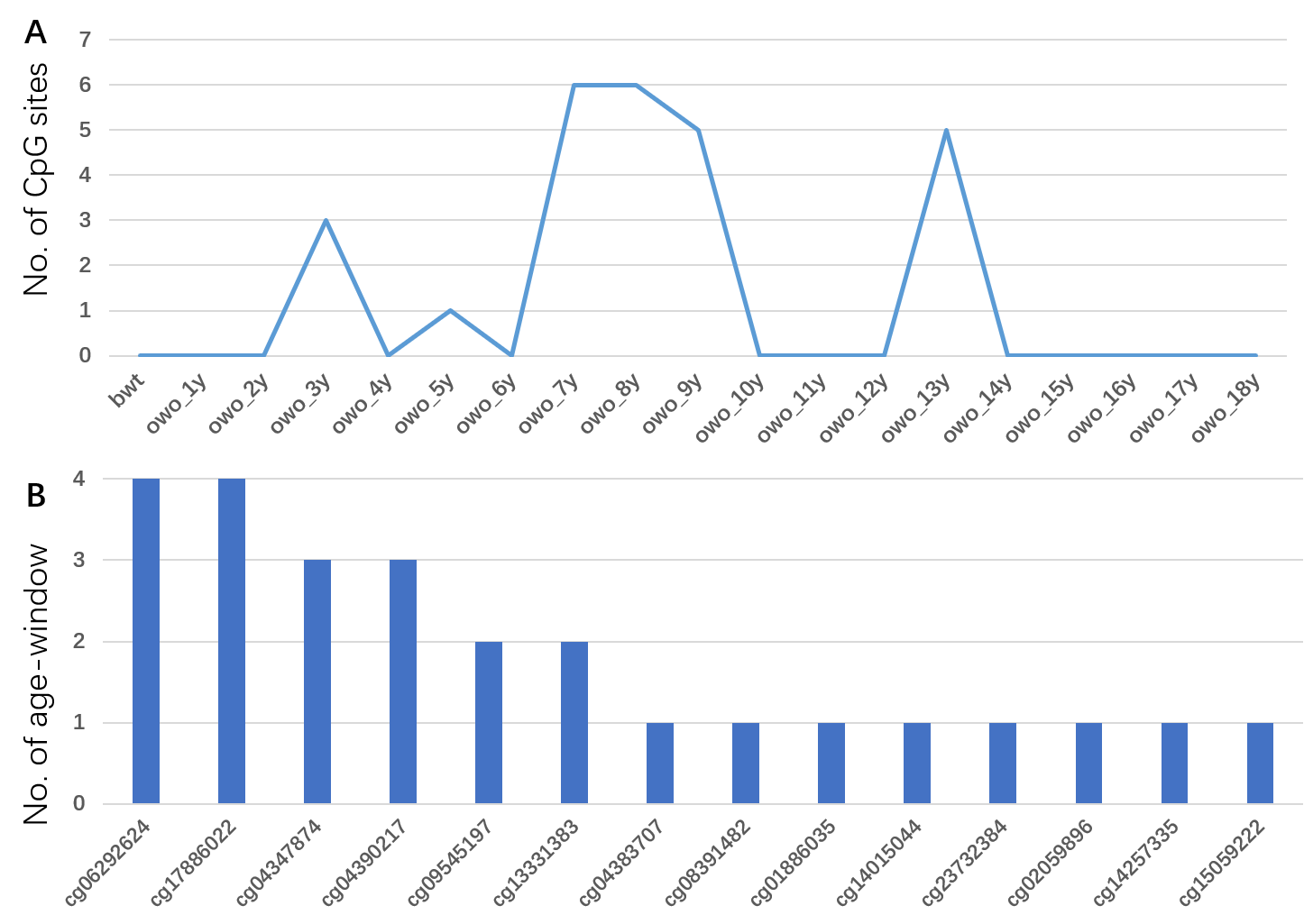
Additional file 2: Fig. S2. Number of CpG sites with significant mediation effect on the maternal BMI-newborn birthweight and maternal BMI-child overweight or obesity at age 1-18 years association in 903 mother-child pairs for each age-window outcome (A); Number of single age-window outcomes showed significant mediation effect for each CpG site (B).
